# How to analyze work productivity loss due to health problems in randomized controlled trials? A simulation study

**DOI:** 10.1186/s12874-021-01330-w

**Published:** 2021-06-24

**Authors:** Wei Zhang, Huiying Sun

**Affiliations:** 1 School of Population and Public Health, University of British Columbia, Vancouver, British Columbia Canada; 2 Centre for Health Evaluation and Outcome Sciences, 588-1081 Burrard Street, Vancouver, British Columbia V6Z 1Y6 Canada

**Keywords:** Productivity loss, Absenteeism, Presenteeism, Zero-inflated data, Simulation studies, Randomized controlled trial, Two-part model, Three-part model

## Abstract

**Background:**

An increasing number of randomized controlled trials (RCTs) have measured the impact of interventions on work productivity loss. Productivity loss outcome is inflated at zero and max loss values. Our study was to compare the performance of five commonly used methods in analysis of productivity loss outcomes in RCTs.

**Methods:**

We conducted a simulation study to compare Ordinary Least Squares (OLS), Negative Binominal (NB), two-part models (the non-zero part following truncated NB distribution or gamma distribution) and three-part model (the middle part between zero and max values following Beta distribution). The main number of observations each arm, N_obs_, that we considered were 50, 100 and 200. Baseline productivity loss was included as a covariate.

**Results:**

All models performed similarly well when baseline productivity loss was set at the mean value. When baseline productivity loss was set at other values and N_obs_ = 50 with ≤5 subjects having max loss, two-part models performed best if the proportion of zero loss> 50% in at least one arm and otherwise, OLS performed best. When N_obs_ = 100 or 200, the three-part model performed best if the two arms had equal scale parameters for their productivity loss outcome distributions between zero and max values.

**Conclusions:**

Our findings suggest that when treatment effect at any given values of one single covariate is of interest, the model selection depends on the sample size, the proportions of zero loss and max loss, and the scale parameter for the productivity loss outcome distribution between zero and max loss in each arm of RCTs.

**Supplementary Information:**

The online version contains supplementary material available at 10.1186/s12874-021-01330-w.

## Background

In addition to direct medical care costs, indirect cost (i.e., work productivity loss due to health problems) is an important component when estimating burden of illness [1–4] or conducting economic evaluations of health care interventions from a societal perspective [5, 6]. Correspondingly, in recent years, an increasing number of randomized controlled trials (RCTs) have measured the impact of health care interventions on work productivity loss either considering it as one patient-centered outcome [7–14] or as one component for economic evaluations [15–18].

Complete components of work productivity loss include 1) absenteeism; 2) presenteeism; 3) employment status changes including reduced routine work hours and work stoppage due to illness [19]. Before transformed into monetary amount, work productivity loss due to health is usually first expressed as work time loss, i.e., counting the days missed from work (absenteeism), the hours lost due to reduced productivity while working (presenteeism), or stopped workdays. Therefore, productivity loss data could be non-negative count data. When presenteeism is first measured using percentage of loss (e.g., from the Work Productivity and Activity Impairment Questionnaire (WPAI) [20, 21] and the Valuation of Lost Productivity questionnaire [22, 23]) and then transformed into work time loss by multiplying the percentage of loss by the actual working time, the estimate may also be non-negative continuous data.

When estimating work time loss among a study population, people can be divided into three groups: I) those with no time loss, II) those with some time loss, and III) those who have lost all work time. Studies have shown that the proportion of Group I is very high, i.e., zero loss [24–27]. Such a distribution applies to the sum of time loss from all the three subcomponents and the time loss from absenteeism and presenteeism, respectively. For example, among patients with arthritis, a study found that the frequency of ‘0’ value varied by the measurement questionnaires: 61% if presenteeism was measured using the Health Labour Questionnaire, 5% using Work Limitations Questionnaire, 16% using the World Health Organization Health and Work Performance Questionnaire, and 27% using WPAI [25]. A clinical trial among employed patients with early rheumatoid arthritis showed that about 50–70% did not have any paid work productivity loss (sum of all three subcomponents) depending on the follow-up time point, and that about 5–10% stopped working due to health problems at Week 13 or cumulatively at Week 52 [27], which contributed to a high proportion of Group I and Group III, i.e., inflated zero value and maximum value.

Various statistical models have been used for analyzing productivity loss. Ordinary Least Squares (OLS) regression was commonly used in previous studies [7–9, 28, 29]. Poisson and Negative Binomial (NB) models for count data were also used [11, 12]. Some studies avoided estimating the mean time loss by using logistic models where productivity loss was treated as binary or categorical variables [10, 30, 31]. These methods may be problematic. For example, the logistic models did not take full use of the continuous data information. Various models have been suggested to deal with zero-inflated and bound-inflated data, e.g., two-part models, zero-inflated models, and other mixture models [32–36]. Kleinman et al. utilized a two-part model to estimate annual lost days due to absenteeism and presenteeism [37]. Also, zero-inflated models were used to analyze productivity loss outcomes [13, 27]. Each method has its own assumptions and its estimation could be biased if these assumptions are not satisfied. Thus, it is important to compare which analytic method works best for analyzing work productivity loss. This study’s objective was to compare the performance of these commonly used methods for analyzing work time loss data in RCTs using simulations.

## Methods

Our simulation methods followed the published guidance by Morris et al. [38] on using simulation studies to evaluate statistical methods.

### Data-generating mechanisms

#### Distributions of productivity loss outcome Y

We assumed that the productivity (time) loss outcome *Y* in an RCT depends on the treatment *arm* (*arm* = 0, 1) and a covariate *x*, denoted by *Y*(*arm*, *x*). For given *arm* and a value of *x*, the probabilities of *Y*(*arm*, *x*) being zero (Group I above), all loss *Max* (Group III), and in (0, *Max*) (Group II) are denoted by *P*_1_(*arm*, *x*), *P*_3_(*arm*, *x*), and *P*_2_(*arm*, *x*), respectively, where *P*_1_(*arm*, *x*) + *P*_2_(*arm*, *x*) + *P*_3_(*arm*, *x*) = 1. The relationships among treatment *arm*, covariate *x* and the probabilities *P*_1_(*arm*, *x*), *P*_2_(*arm*, *x*), and *P*_3_(*arm*, *x*) are given by the follow equations:

$$ {P}_1\left( arm,x\right)=\frac{\exp \left({\alpha}_1+{\beta}_1 arm+{\gamma}_1x\right)}{\exp \left({\alpha}_1+{\beta}_1 arm+{\gamma}_1x\right)+\exp \left({\alpha}_2+{\beta}_2 arm+{\gamma}_2x\right)+1} $$

$$ {P}_2\left( arm,x\right)=\frac{\exp \left({\alpha}_2+{\beta}_2 arm+{\gamma}_2x\right)}{\exp \left({\alpha}_1+{\beta}_1 arm+{\gamma}_1x\right)+\exp \left({\alpha}_2+{\beta}_2 arm+{\gamma}_2x\right)+1} $$

$$ {P}_3\left( arm,x\right)=\frac{1}{\exp \left({\alpha}_1+{\beta}_1 arm+{\gamma}_1x\right)+\exp \left({\alpha}_2+{\beta}_2 arm+{\gamma}_2x\right)+1} $$

where *α*_1_, *β*_1_, *γ*_1_, *α*_2_, *β*_2_, and *γ*_2_ are given parameters at each simulation.

We denoted *Y*(*arm*, *x*) truncated at 0 and *Max* by ${\left.Y\left( arm,x\right)\right|}_0^{Max}$. We assumed that ${\left.Y\left( arm,x\right)\right|}_0^{max}$ follows a truncated NB distribution, denoted by ${\left. NB\left(r\left( arm,x\right),p\Big( arm,x\right)\Big)\right|}_0^{Max}$, with mean *E*_*r*(*arm*, *x*), *p*(*arm*, *x*)_{*Y*(*arm*, *x*)| 0 < *Y*(*arm*, *x*) < *Max*} and standard deviation *SD*_*r*(*arm*, *x*), *p*(*arm*, *x*)_{*Y*(*arm*, *x*)| 0 < *Y*(*arm*, *x*) < *Max*}. We further assumed that

$$ {E}_{r\left( arm,x\right),p\left( arm,x\right)}\left\{Y\left( arm,x\right)|0<Y\left( arm,x\right)<\mathit{\operatorname{Max}}\right\}={E}_{r\left( arm,0\right),p\left( arm,0\right)}\left\{Y\left( arm,0\right)|0<Y\left( arm,0\right)<\mathit{\operatorname{Max}}\right\}+a\cdotp x $$

and *p*(*arm*, *x*) = *p*(*arm*, 0) for all *x*, where *a* is a parameter assumed. Thus, for given *E*_*r*(*arm*, 0), *p*(*arm*, 0)_{*Y*(*arm*, 0)| 0 < *Y*(*arm*, 0) < *Max*}, *SD*_*r*(*arm*, 0), *p*(*arm*, 0)_{*Y*(a*rm*, 0)| 0 < *Y*(*arm*, 0) < *Max*}, and parameter *a* at each simulation, the NB parameters *r*(*arm*, *x*) and *p*(*arm*, *x*) can be derived for given *arm* and *x* (see the detailed derivation in Additional file 1).

For given *arm* and *x*, the mean of the productivity loss outcome *Y*(*arm*, *x*) can be calculated by

$$ E\left\{Y\left( arm,x\right)\right\}={P}_1\left( arm,x\right)\cdotp 0+{P}_2\left( arm,x\right)\cdotp {E}_{r\left( arm,x\right),p\left( arm,x\right)}\left\{Y\left( arm,x\right)|0<Y\left( arm,x\right)<\mathit{\operatorname{Max}}\right\}+{P}_3\left( arm,x\right)\cdotp \mathit{\operatorname{Max}} $$

#### Distribution for covariate x

Since most RCTs are not randomized by patients’ productivity loss at baseline, which is highly correlated with productivity loss outcome, it is common to use regression models to adjust for baseline productivity loss. Therefore, in this study, we assumed *x* to be the productivity loss at baseline which follows a distribution with *P*, the probability of being zeros (*P* > 0), and 1 – *P*, the probability being non-zero values from a NB distribution truncated at 0 and *Max*, ${\left. NB\left({r}_x,{p}_x\right)\right|}_0^{Max}$. That is, *x* is independent of treatments and all RCT participants should be working at baseline and thus their productivity loss does not equal to *Max*. The truncated NB distribution has mean = *μ*_*x*_ and variance = *sd*_*x*_. At each simulation, *μ*_*x*_ and *sd*_*x*_ are given parameters and *r*_*x*_ and *p*_*x*_ are derived from *μ*_*x*_ and *sd*_*x*_.

#### Simulation parameters

We assumed the time period for estimating work productivity loss is 12 weeks and the maximum loss time is 60 days (*Max* = 60). We considered three sets of parameters for the multinomial distributions for *Y*(*arm*, *x*), three sets of parameters for ${\left. NB\left(r\left( arm,x\right),p\Big( arm,x\Big)\right)\right|}_0^{60}$ and one set of parameters for baseline productivity loss *x* (see all parameters in Table 1). The parameters were chosen based on our review of recently published articles which measured absenteeism and presenteeism in an RCT [7–14, 27, 39].

**Table 1:** Parameters in the simulation study

Parameters	80:15:5/60:30:10	60:35:5/40:50:10	50:40:10/30:55:15
Number of observations each arm: *N*_*obs*_	50; 100; 200	50; 100; 200	50; 100; 200
**Multinomial distribution of productivity loss outcomes**			
Probability of zero loss: *P*_1_(*arm*, 0)			
Arm 1	0.80	0.60	0.50
Arm 0	0.60	0.40	0.30
Probability of loss between 0 and *Max*: *P*_2_(*arm*, 0)			
Arm 1	0.15	0.35	0.40
Arm 0	0.30	0.50	0.55
Probability of max loss: *P*_3_(*arm*, 0)			
Arm 1	0.05	0.05	0.10
Arm 0	0.10	0.10	0.15
Coefficient of *x*			
*γ*_1_	−0.02	−0.02	−0.02
*γ*_2_	−0.01	−0.01	− 0.01
**Truncated negative binomial distribution of productivity loss outcomes between 0 and** ***Max***			
Mean: *E* for Arm 1	15.00	15.00	15.00
Mean: *E* for Arm 0	20.00	20.00	20.00
*Max* loss	60.00	60.00	60.00
Equal scale			
*SD* for Arm 1	13.34	13.34	13.34
*SD* for Arm 0	14.63	14.63	14.63
Unequal scale			
*SD* for Arm 1	12.00	12.00	12.00
*SD* for Arm 0	16.00	16.00	16.00
Coefficient of *x*: *a*	0.15	0.15	0.15
**Baseline productivity loss**			
Probability of zero loss: *P*	0.30	0.30	0.30
Mean for non-zero loss: *μ*_*x*_	20.00	20.00	20.00
SD for non-zero loss: *sd*_*x*_	16.00	16.00	16.00

*SD* standard deviation

#### Number of observations each arm N_obs_

Similarly, based on the common sample size in the previous RCT studies, we chose *N*_*obs*_ = 50, 100, and 200 participants who are working at baseline for each arm at each simulation. A sample size of 1000 and 2000 were also used to check whether bias (see definition below) varied by sample size.

#### Simulation algorithm

At each simulation, we generated *N*_*obs*_ samples for each *arm* in the following steps.

For *arm* = 0, 1 and *i* = 1, 2, 3, ……*N*_*obs*_,

1. Randomly generate *b*_*i*_ from Bernoulli distribution Bernoulli (*P*). If *b*_*i*_ = 1, then let *x*_*i*_ = 0. If *b*_*i*_ = 0, then randomly generate *x*_*i*_ from ${\left. NB\left({r}_x,{p}_x\right)\right|}_0^{60}$.
2. Randomly generate vector (*K*_1_(*arm*, *x*_*i*_), *K*_2_(*arm*, *x*_*i*_), *K*_3_(*arm*, *x*_*i*_)) from the multinomial distribution (*P*_1_(*arm*, *x*_*i*_), *P*_2_(*arm*, *x*_*i*_), *P*_3_(*arm*, *x*_*i*_),1), where *K*_1_(*arm*, *x*_*i*_), *K*_2_(*arm*, *x*_*i*_), and *K*_3_(*arm*, *x*_*i*_) are either 0 or 1 and ${\sum}_{j=1}^3{K}_j\left( arm,{x}_i\right)=1$.
3. Randomly generate *Z*(*arm*, *x*_*i*_) from ${\left. NB\left(r\left( arm,{x}_i\right),p\Big( arm,{x}_i\right)\Big)\right|}_0^{Max}.$
4. The productivity loss outcome *Y*_*i*_(*arm*) is defined by
  $$ {Y}_i(arm)=\left\{\begin{array}{c}0\  if\ {K}_1\left( arm,{x}_i\right)=1\\ {}Z\left( arm,{x}_i\right)\  if\ {K}_2\left( arm,{x}_i\right)=1\ \\ {}60\  if\ {K}_3\left( arm,{x}_i\right)=1\ \end{array}\right. $$

### Estimation methods

Each simulated dataset was analyzed using the following five regression models:

1. OLS;
2. NB: generalized linear model for NB distribution;
3. ZTNB: two-part model – logistic regression for the probability of being zero, and generalized linear regression with zero-truncated NB distribution (Hurdle) for the non-zeros.
4. ZG: two-part model – logistic regression for the probability of being zero, and generalized linear regression with Gamma distribution for the non-zeros;
5. Three-part model: multinomial logistic regression for the probabilities of being zero and 60 and generalized linear regression with Beta distribution for the those with values in (0, 60) (transformed to (0, 1)).

### Estimand

Our estimands in the simulation study were *θ*(*x*) = *E*{*Y*(1, *x*)} − *E*{*Y*(0, *x*)} at the values of *x* of interest. The estimates of estimand *θ*(*x*) in each regression, denoted by $\hat{\theta}(x),$ were derived from the given *x* and the estimated regression parameters. We used bootstrapping method, 1000 replications, to estimate the standard error (SE) of $\hat{\theta}(x)$ in all regressions except OLS.

### Performance measures

Our key performance measure of interest was bias $\hat{\theta}(x)-\theta (x)$. We assumed $SD\left(\hat{\theta}(x)\right)\le 4$ (the standard deviation of the estimators $\hat{\theta}(x)$) and considered 0.08 as an acceptable Monte Carlo SE of bias. Accordingly, we needed at least 2500 repetitions based on the published simulation study guidance [38]. We chose our final number of repetitions, *n*_*sim*_ = 5000 and thus Monte Carlo SE of coverage would be 0.3 and 0.7 for coverage of 95 and 50%, respectively, which are acceptable.

Our performance measures included bias, coverage, power, empirical and model-based SE, and the mean squared error (MSE) for $\hat{\theta}(x)$ at *x* = *μ*_*x*_, 0 and 30. Their definitions can be found in Morris et al. [38]. All analyses were performed using SAS 9.4. SAS codes used for our simulation study are available in Additional file 1.

## Results

### Computational issues

We encountered convergence issues mainly in the scenarios with smaller number of observations and higher proportion of Group I and lower proportion of Group III. For example, when *N*_*obs*_ =50, the simulated databases based on 80% zero loss could not generate enough samples for Group II and Group III and thus we did not compare the five models in this scenario. Similarly, the simulated databases based on 5% max loss could have no samples for Group III and thus the three-part model was considered for such scenarios. For some of the remaining scenarios, the issue of quasi-complete separation (the maximum likelihood estimate may not exist) was detected while running multinomial logistic regression for the three-part model. Table 2 presents the number of databases with quasi-complete separation detected among the 5000 simulated databases by three different sets of distributions of productivity loss outcomes in two arms (i.e., the proportions of zero loss, some loss and max loss for productivity loss outcomes in the two arms); *N*_*obs*_ = 50, 100 and 200; and whether the two arms have equal scale parameters for truncated NB distributions of their productivity loss outcomes. The number of simulations with the convergence issue was very small.

**Table 2:** Number of simulated databases with quasi-complete separation

Distribution of productivity loss outcome	Number of Observations in each arm	Truncated negative binomial distributions of productivity loss outcomes in the two arms	Number of databases with quasi-complete separation^**a**^
80:15:5/60:30:10	100	Equal Scale	3
80:15:5/60:30:10	100	Unequal Scale	7
80:15:5/60:30:10	200	Equal Scale	0
80:15:5/60:30:10	200	Unequal Scale	0
60:35:5/40:50:10	100	Equal Scale	0
60:35:5/40:50:10	100	Unequal Scale	0
60:35:5/40:50:10	200	Equal Scale	0
60:35:5/40:50:10	200	Unequal Scale	0
50:40:10/30:55:15	50	Equal Scale	0
50:40:10/30:55:15	50	Unequal Scale	2
50:40:10/30:55:15	100	Equal Scale	0
50:40:10/30:55:15	100	Unequal Scale	0
50:40:10/30:55:15	200	Equal Scale	0
50:40:10/30:55:15	200	Unequal Scale	0

^a^The maximum likelihood estimate may not exist while running multinomial logistic regression for the three-part models. For number of observations = 50 and 5% max loss, a large number of databases with quasi-separation and thus the three-part model was not considered for these scenarios

Table 3 presents the proportions of the total 10,000 simulations (5000 for equal scale and 5000 for unequal scale) that have the number of quasi-complete separation detected among their 1000 bootstraps equal to 0, 1–10, 11–100, 101–400, 401–700, 701–950, and 950–1000 by the distributions of productivity loss outcomes and *N*_*obs*_. The issue was the most problematic in the scenario with the distribution of productivity loss outcome, 80%/15%/5% vs. 60%/30%/10%, and 100 observations. Only 38.03% of the 10,000 simulations did not have any bootstraps with quasi-complete separation, 41.97% had 1–10 bootstraps with quasi-complete separation and 0.09% had > 950 bootstraps with quasi-complete separation.

**Table 3:** The proportions of simulations that having quasi-complete separation issues among their 1000 bootstraps

Distribution of productivity loss outcome	Number of observations in each arm	Number of quasi-complete separation detected in 1000 bootstraps						
		0	1–10	11–100	101–400	401–700	701–950	> 950
80:15:5/60:30:10	100	38.03	41.97	16.64	3.25	0.01	0.01	0.09
80:15:5/60:30:10	200	94.75	5.00	0.24	0.01	0	0	0
60:35:5/40:50:10	100	61.16	32.83	5.63	0.38	0	0	0
60:35:5/40:50:10	200	99.17	0.81	0.02	0	0	0	0
50:40:10/30:55:15	50	39.45	45.05	14.30	1.11	0.08	0.01	0
50:40:10/30:55:15	100	97.46	2.46	0.08	0	0	0	0
50:40:10/30:55:15	200	100	0	0	0	0	0	0

Among 10000 simulations for each row: 5000 for equal scale and 5000 for unequal scale for the truncated negative binomial distributions of productivity loss outcomes in the two arms

### Bias

**Fig. 1:**
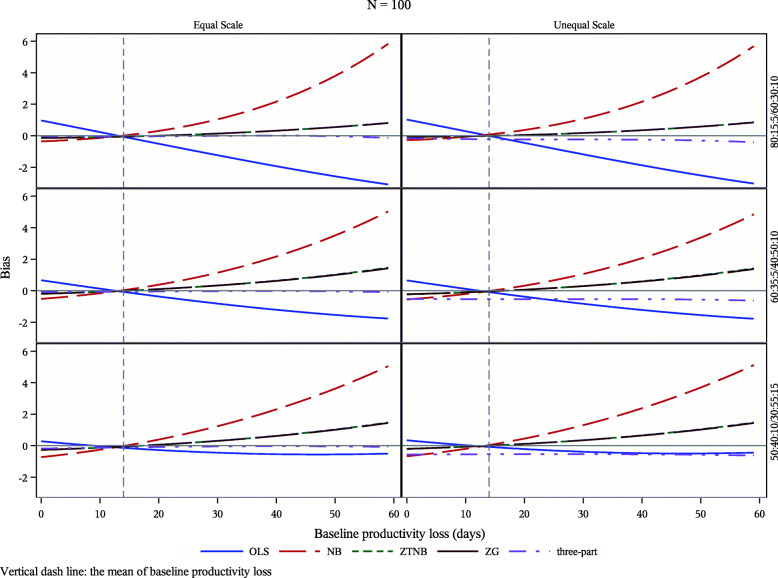
Mean bias for the number of observations in each arm = 100. Legends: OLS: ordinary least squares; NB: negative binomial; ZTNB: two-part model – logistic regression for the probability of being zero, and generalized linear regression with zero-truncated NB distribution for the non-zeros; ZG: two-part model – logistic regression for the probability of being zero, and generalized linear regression with Gamma distribution for the non-zeros; Three-part: multinomial logistic regression for the probabilities of being zero and 60 and generalized linear regression with Beta distribution for the those with values in (0, 60) (transformed to (0, 1))

Figure 1 (for *N*_*obs*_ = 100) and Supplementary Figure S1 (*N*_*obs*_ = 50), S2 (*N*_*obs*_ = 200), S3 (*N*_*obs*_ = 1000) and S4 (*N*_*obs*_ = 2000) (see Additional file 2) present the bias for the five regression models by the value of baseline productivity loss, *x*; three different sets of distributions of productivity loss outcomes in two arms; and whether the two arms have equal scale parameters for truncated NB distributions of their productivity loss outcomes.

#### Covariate x

All models performed similarly well at the mean of baseline productivity loss, *x* = 14. At other assessed *x* values, NB performed worst for all scenarios and three-part model performed best. Two-part models performed almost exactly the same for any *x* values regardless whether the second part was assumed to be a truncated NB or gamma distribution.

#### Proportions of zero loss and max loss

When productivity loss outcome had a high proportion of zero loss (> 50% in at least one arm) and a non-trivial proportion of max loss (> 5%), two-part models and three-part model performed better than OLS and NB models. The performance of OLS was getting better when the proportion of zero loss was lower (≤50% in both arms) and could be better than two-part models when *x* values were higher.

#### Different scale parameters between the two arms

When the two arms had unequal scale parameters in the assumed truncated NB distributions for their productivity loss outcomes, three-part model provided bias estimators for all values of *x*. Two-part models could produce lower bias than three-part model at lower values of *x*. OLS performed best or as well as two-part models or three-part model in the databases with the proportions of productivity loss outcomes at 50%/40%/10% vs. 30%/55%/15%.

#### Sample size

For *N*_*obs*_ = 50, we only applied the three-part model for databases with the proportions of productivity loss outcomes at 50%/40%/10% vs. 30%/55%/15%. The performance results based on bias did not change when *N*_*obs*_ increased from 50 to 100, 200, 1000 and 2000.

### Other performance measures

Similar to bias, other performance measures, including coverage, power, empirical SE, model SE and MSE, were comparable among the five different models when *x* = 14 (Tables 4, 5 and 6 and Supplementary Tables S1-S3 in Additional file 3). However, when *x* = 0 or 30, the coverages of the two-part models and three-part model were similar; the coverage of the three-part model was slightly larger than that of OLS and NB except when the proportions of productivity loss outcomes were at 50%/40%/10% vs. 30%/55%/15% (Table 4 and Supplementary Tables S1-S3 in Additional file 3). When *x* = 0, the empirical SE and MSE of the NB, two-part models and three-part model were similar and they were all lower than those of OLS in all scenarios (Tables 5 and 6 and Supplementary Tables S1-S3 in Additional file 3). On the other hand, when *x* = 30, the empirical SE and MSE of OLS were the lowest, followed by the three-part model. Those of NB were the highest. The two-part models (ZTNB and ZG) performed the same using these performance measures.

**Table 4:** Coverage for the number of observations each arm = 100

Distribution of productivity loss outcome	Scales in the two arms^**a**^	OLS	NB	ZTNB	ZG	Three-part
***x*** **= mean**						
80:15:5/60:30:10	Equal Scale	95.2% (0.003)	94.7% (0.003)	94.5% (0.003)	94.5% (0.003)	94.6% (0.003)
80:15:5/60:30:10	Unequal Scale	94.7% (0.003)	94.4% (0.003)	94.3% (0.003)	94.3% (0.003)	94.4% (0.003)
60:35:5/40:50:10	Equal Scale	95.2% (0.003)	94.7% (0.003)	94.8% (0.003)	94.7% (0.003)	94.8% (0.003)
60:35:5/40:50:10	Unequal Scale	94.5% (0.003)	94.3% (0.003)	94.3% (0.003)	94.3% (0.003)	94.1% (0.003)
50:40:10/30:55:15	Equal Scale	95.2% (0.003)	95.0% (0.003)	94.9% (0.003)	94.9% (0.003)	95.2% (0.003)
50:40:10/30:55:15	Unequal Scale	95.4% (0.003)	94.9% (0.003)	94.8% (0.003)	94.8% (0.003)	94.8% (0.003)
***x*** **= 0**						
80:15:5/60:30:10	Equal Scale	93.4% (0.004)	94.3% (0.003)	94.7% (0.003)	94.7% (0.003)	95.0% (0.003)
80:15:5/60:30:10	Unequal Scale	93.1% (0.004)	93.9% (0.003)	94.2% (0.003)	94.2% (0.003)	94.2% (0.003)
60:35:5/40:50:10	Equal Scale	94.2% (0.003)	94.3% (0.003)	94.4% (0.003)	94.4% (0.003)	95.1% (0.003)
60:35:5/40:50:10	Unequal Scale	94.3% (0.003)	93.6% (0.003)	94.3% (0.003)	94.3% (0.003)	94.4% (0.003)
50:40:10/30:55:15	Equal Scale	95.2% (0.003)	93.9% (0.003)	94.9% (0.003)	94.8% (0.003)	95.1% (0.003)
50:40:10/30:55:15	Unequal Scale	95.0% (0.003)	93.9% (0.003)	94.8% (0.003)	94.8% (0.003)	94.7% (0.003)
***x*** **= 30**						
80:15:5/60:30:10	Equal Scale	92.6% (0.004)	94.1% (0.003)	94.5% (0.003)	94.5% (0.003)	94.6% (0.003)
80:15:5/60:30:10	Unequal Scale	92.0% (0.004)	93.7% (0.003)	94.3% (0.003)	94.3% (0.003)	94.4% (0.003)
60:35:5/40:50:10	Equal Scale	94.8% (0.003)	93.5% (0.003)	94.8% (0.003)	94.7% (0.003)	95.3% (0.003)
60:35:5/40:50:10	Unequal Scale	93.9% (0.003)	93.4% (0.004)	94.3% (0.003)	94.3% (0.003)	94.6% (0.003)
50:40:10/30:55:15	Equal Scale	95.1% (0.003)	94.0% (0.003)	95.0% (0.003)	94.9% (0.003)	95.4% (0.003)
50:40:10/30:55:15	Unequal Scale	95.2% (0.003)	93.5% (0.003)	94.8% (0.003)	94.8% (0.003)	95.0% (0.003)

*OLS* ordinary least squares, *NB* negative binomial, *ZTNB* two-part model – logistic regression for the probability of being zero, and generalized linear regression with zero-truncated NB distribution for the non-zeros, *ZG* two-part model – logistic regression for the probability of being zero, and generalized linear regression with Gamma distribution for the non-zeros; Three-part: multinomial logistic regression for the probabilities of being zero and 60 and generalized linear regression with Beta distribution for the those with values in (0, 60) (transformed to (0, 1))

^a^for truncated negative binomial distributions of productivity loss outcomes in the two arms

**Table 5:** Empirical standard error for the number of observations each arm = 100

Distribution of productivity loss outcome	Scales in the two arms^**a**^	OLS	NB	ZTNB	ZG	Three-part
***x*** **= mean**						
80:15:5/60:30:10	Equal Scale	2.708 (0.027)	2.813 (0.028)	2.714 (0.027)	2.716 (0.027)	2.677 (0.027)
80:15:5/60:30:10	Unequal Scale	2.756 (0.028)	2.867 (0.029)	2.766 (0.028)	2.768 (0.028)	2.718 (0.027)
60:35:5/40:50:10	Equal Scale	2.734 (0.027)	2.793 (0.028)	2.750 (0.028)	2.752 (0.028)	2.671 (0.027)
60:35:5/40:50:10	Unequal Scale	2.744 (0.027)	2.806 (0.028)	2.761 (0.028)	2.763 (0.028)	2.689 (0.027)
50:40:10/30:55:15	Equal Scale	3.025 (0.030)	3.096 (0.031)	3.056 (0.031)	3.058 (0.031)	2.981 (0.030)
50:40:10/30:55:15	Unequal Scale	3.051 (0.031)	3.119 (0.031)	3.082 (0.031)	3.084 (0.031)	3.013 (0.030)
***x*** **= 0**						
80:15:5/60:30:10	Equal Scale	2.708 (0.027)	2.447 (0.024)	2.414 (0.024)	2.416 (0.024)	2.415 (0.024)
80:15:5/60:30:10	Unequal Scale	2.756 (0.028)	2.499 (0.025)	2.465 (0.025)	2.467 (0.025)	2.456 (0.025)
60:35:5/40:50:10	Equal Scale	2.734 (0.027)	2.448 (0.024)	2.495 (0.025)	2.498 (0.025)	2.504 (0.025)
60:35:5/40:50:10	Unequal Scale	2.744 (0.027)	2.458 (0.025)	2.489 (0.025)	2.492 (0.025)	2.497 (0.025)
50:40:10/30:55:15	Equal Scale	3.025 (0.030)	2.711 (0.027)	2.779 (0.028)	2.782 (0.028)	2.781 (0.028)
50:40:10/30:55:15	Unequal Scale	3.051 (0.031)	2.746 (0.027)	2.818 (0.028)	2.822 (0.028)	2.827 (0.028)
***x*** **= 30**						
80:15:5/60:30:10	Equal Scale	2.708 (0.027)	4.118 (0.041)	3.346 (0.033)	3.348 (0.033)	3.234 (0.032)
80:15:5/60:30:10	Unequal Scale	2.756 (0.028)	4.100 (0.041)	3.388 (0.034)	3.390 (0.034)	3.266 (0.033)
60:35:5/40:50:10	Equal Scale	2.734 (0.027)	3.613 (0.036)	3.228 (0.032)	3.227 (0.032)	3.019 (0.030)
60:35:5/40:50:10	Unequal Scale	2.744 (0.027)	3.623 (0.036)	3.252 (0.033)	3.250 (0.032)	3.060 (0.031)
50:40:10/30:55:15	Equal Scale	3.025 (0.030)	3.847 (0.038)	3.518 (0.035)	3.516 (0.035)	3.265 (0.033)
50:40:10/30:55:15	Unequal Scale	3.051 (0.031)	3.841 (0.038)	3.523 (0.035)	3.520 (0.035)	3.281 (0.033)

*OLS* ordinary least squares, *NB* negative binomial, *ZTNB* two-part model – logistic regression for the probability of being zero, and generalized linear regression with zero-truncated NB distribution for the non-zeros, *ZG* two-part model – logistic regression for the probability of being zero, and generalized linear regression with Gamma distribution for the non-zeros; Three-part: multinomial logistic regression for the probabilities of being zero and 60 and generalized linear regression with Beta distribution for the those with values in (0, 60) (transformed to (0, 1))

^a^for truncated negative binomial distributions of productivity loss outcomes in the two arms

**Table 6:** Mean squared error for the number of observations each arm = 100

Distribution of productivity loss outcome	Scales in the two arms^**a**^	OLS	NB	ZTNB	ZG	Three-part
***x*** **= mean**						
80:15:5/60:30:10	Equal Scale	7.337 (0.146)	7.912 (0.163)	7.369 (0.148)	7.379 (0.148)	7.168 (0.145)
80:15:5/60:30:10	Unequal Scale	7.596 (0.152)	8.221 (0.168)	7.648 (0.153)	7.659 (0.153)	7.434 (0.148)
60:35:5/40:50:10	Equal Scale	7.477 (0.145)	7.800 (0.153)	7.562 (0.147)	7.573 (0.147)	7.139 (0.139)
60:35:5/40:50:10	Unequal Scale	7.538 (0.153)	7.872 (0.157)	7.625 (0.153)	7.636 (0.153)	7.519 (0.149)
50:40:10/30:55:15	Equal Scale	9.167 (0.181)	9.585 (0.190)	9.341 (0.185)	9.355 (0.185)	8.898 (0.175)
50:40:10/30:55:15	Unequal Scale	9.313 (0.182)	9.726 (0.188)	9.494 (0.183)	9.507 (0.184)	9.369 (0.183)
***x*** **= 0**						
80:15:5/60:30:10	Equal Scale	8.273 (0.164)	6.117 (0.140)	5.846 (0.121)	5.853 (0.121)	5.832 (0.123)
80:15:5/60:30:10	Unequal Scale	8.649 (0.175)	6.323 (0.165)	6.081 (0.124)	6.090 (0.125)	6.053 (0.123)
60:35:5/40:50:10	Equal Scale	7.917 (0.153)	6.260 (0.123)	6.266 (0.123)	6.275 (0.123)	6.275 (0.127)
60:35:5/40:50:10	Unequal Scale	7.960 (0.163)	6.345 (0.129)	6.250 (0.127)	6.259 (0.127)	6.502 (0.131)
50:40:10/30:55:15	Equal Scale	9.228 (0.180)	7.873 (0.158)	7.795 (0.156)	7.811 (0.156)	7.765 (0.156)
50:40:10/30:55:15	Unequal Scale	9.426 (0.184)	7.988 (0.155)	7.985 (0.155)	8.001 (0.156)	8.305 (0.162)
***x*** **= 30**						
80:15:5/60:30:10	Equal Scale	8.865 (0.173)	18.038 (0.564)	11.210 (0.231)	11.228 (0.232)	10.454 (0.215)
80:15:5/60:30:10	Unequal Scale	8.991 (0.174)	17.965 (0.493)	11.506 (0.238)	11.519 (0.238)	10.718 (0.215)
60:35:5/40:50:10	Equal Scale	8.139 (0.159)	14.363 (0.326)	10.529 (0.212)	10.519 (0.212)	9.113 (0.178)
60:35:5/40:50:10	Unequal Scale	8.219 (0.163)	14.250 (0.322)	10.661 (0.220)	10.643 (0.220)	9.646 (0.196)
50:40:10/30:55:15	Equal Scale	9.347 (0.187)	16.324 (0.365)	12.471 (0.253)	12.456 (0.253)	10.663 (0.211)
50:40:10/30:55:15	Unequal Scale	9.455 (0.185)	16.445 (0.347)	12.530 (0.247)	12.511 (0.247)	11.045 (0.218)

*OLS* ordinary least squares, *NB* negative binomial, *ZTNB* two-part model – logistic regression for the probability of being zero, and generalized linear regression with zero-truncated NB distribution for the non-zeros, *ZG* two-part model – logistic regression for the probability of being zero, and generalized linear regression with Gamma distribution for the non-zeros; Three-part: multinomial logistic regression for the probabilities of being zero and 60 and generalized linear regression with Beta distribution for the those with values in (0, 60) (transformed to (0, 1))

^a^for truncated negative binomial distributions of productivity loss outcomes in the two arms

## Discussion

In this paper, we compared different statistical models for analyzing productivity loss outcomes in RCTs by considering their data distribution characteristics with inflated zero and max values. We focused on five commonly used models, OLS, NB, ZTNB, ZG, and three-part model, adjusting for one covariate. From our simulation results, we found that NB performed worst overall. Two-part models assuming the second part following zero-truncated NB (ZTNB) or Gamma distribution (ZG) performed the same in all scenarios. The performance of OLS, two-part models and three-part model varied in different scenarios. Based on our results, we provided the following practical recommendations if the treatment effect at any given values of one single covariate is of interest:

1. Check the sample size and the proportions of zero loss and max loss in each arm; If the sample size of each arm (i.e., the number of participants who are working at baseline) is ≤50 and there are ≤5 subjects with max loss, three-part model should not be considered. Two-part models (either ZTNB or ZG) should be used if the proportion of zero loss > 50% in at least one arm and OLS should be used if the proportion of zero loss ≤50% in both arms.
2. If the sample size of each arm is > 50 and the proportion of max loss > 5% with more than 5 subjects, then check the scale parameter of the productivity loss outcome distribution between zero and max loss (Group II) for each arm, which was an influencing factor on model performance:
  Our three-part model assumed a Beta distribution for Group II and thus the scale = *α* + *β*, where α and β are Beta distribution parameters derived from the mean and variance of Group II. If the two arms have similar scales, three-part model should be used.
  Otherwise, the two-part models should be used if the proportion of zero loss > 50% in at least one arm and OLS should be used if the proportion of zero loss ≤50% in both arms.

We chose the baseline productivity loss as a single covariate in our simulations, but the above practical recommendations would apply to any single covariate models for analyzing productivity loss outcomes in an RCT, in which the covariate is well balanced between treatment arms and associated with the productivity loss outcomes.

Our study followed the published guidance by Morris et al. [38] for design, execution, analysis, reporting, and presentation of simulation studies. To the best of our knowledge, there have not been simulation studies considering data like productivity loss outcome with excess zero and max values. However, some previous simulation studies have compared different statistical models for data with excess zeros and found that zero inflated models or two-part models performed better than Poisson model, NB model or OLS [34, 35]. Our study showed consistent results that two-part models performed better than OLS if data had > 50% zeros in at least one arm of an RCT.

Our study has limitations. First, as mentioned above, we had convergence issues because of quasi-complete separation in simulated databases or their bootstraps. Thus, we did not apply the three-part model for *N*_*obs*_ =50 in scenarios with 5% max loss. However, for *N*_*obs*_ =100 and 200, the number of simulated databases with quasi-complete separation was very small (Table 2), which should have minimal impact on our mean bias estimates. The quasi-complete separation issues detected in bootstraps were also within an acceptable range (Table 3).

Second, we compared five commonly used statistically methods to make more informative and practical recommendations to a broad clinical audience. However, there are other potential methods that could be used, for example, Poisson model, zero-inflated models and other mixture models. We chose NB model, two-part models and three-part model by assuming they would perform similarly to Poisson model, zero-inflated models and other mixture models, respectively.

Third, our simulation parameters were determined based on published RCTs, which were selected after a rapid literature review of RCT studies that measured and reported work productivity loss (absenteeism or presenteeism or both or all three subcomponents). However, the scenarios considered in our simulation study might not cover all possible scenarios of RCTs. Our simulation method can be used to compare statistical models in other scenarios we did not consider in the future.

## Conclusions

In summary, we conducted a simulation study to compare five statistical models for analyzing productivity loss outcomes in RCTs. Our findings suggest that NB model performs worst. If treatment effect at any given values of a single covariate is of interest, the model selection among OLS, two-part models and three-part model depends on the sample size, the proportions of zero loss and max loss, and the scale of the productivity loss outcome distribution between zero and max loss in each arm of RCTs.

## Supplementary Information

**Additional file 1.** Deviation for the Negative Binomial distribution parameters and SAS codes.

**Additional file 2: Supplementary Figure S1.** Mean bias for the number of observations in each arm = 50. **Supplementary Figure S2.** Mean bias for the number of observations in each arm = 200. **Supplementary Figure S3.** Mean bias for the number of observations in each arm = 1000. **Supplementary Figure S4.** Mean bias for the number of observations in each arm = 2000.

**Additional file 3: Supplementary Table S1.** Performance measures for the number of observations each arm = 50. **Supplementary Table S2.** Performance measures for the number of observations each arm = 100. **Supplementary Table S3.** Performance measures for the number of observations each arm = 200.

## Data Availability

All data generated or analyzed during this study and the corresponding SAS codes are included in this published article and its supplementary information files.

## Abbreviations

MSE: Mean squared error
NB: Negative binomial
OLS: Ordinary least squares
RCT: Randomized controlled trial
SD: Standard deviation
SE: Standard error
WPAI: Work Productivity and Activity Impairment Questionnaire
ZG: Two-part model – logistic regression for the probability of being zero, and generalized linear regression with Gamma distribution for the non-zeros
ZTNB: Two-part model – logistic regression for the probability of being zero, and generalized linear regression with zero-truncated NB distribution (Hurdle) for the non-zeros
