# Supplementary material for: How to analyze work productivity loss due to health problems in randomized controlled trials? A simulation study
Source: BMC Med Res Methodol. 2021 Jun 24;21:130. doi: 10.1186/s12874-021-01330-w (PMC8223308; doi:10.1186/s12874-021-01330-w)
Supplement: Supplementary file 1 — Additional file 1. Deviation for the Negative Binomial distribution parameters and SAS codes. [file 12874_2021_1330_MOESM1_ESM.docx]

**How to analyze work productivity loss due to health problems in randomized controlled trials? A simulation study**

**Supplementary File**

1. **Derivation for the Negative Binomial distribution parameters**

Let $Y$ follows a Negative Binomial distribution $NB\left( r,p \right).$ The mean and variance of $Y$ are given by

$$E_{r,p}\left\{ Y \right\}= r\cdot\frac{1-p}{p}, (1)$$

$${Var}_{r,p}\left\{ Y \right\}= r\cdot\frac{1-p}{p^{2}} . (2)$$

Let $k>0$be an integer. According to Geyer,^1^ the mean and variance of $NB\left( r,p \right)$left-truncated at $k-1$ can be calculated by

$E_{r,p}\left\{ Y|Y\geq k \right\}=E_{r,p}\left\{ Y|Y>k-1 \right\}=r\cdot\frac{1-p}{p}+\frac{k}{p\left( 1+\beta\right)}$ (3)

${Var}_{r,p}\left\{ Y|Y\geq k \right\}={Var}_{r,p}\left\{ Y|Y>k-1 \right\}=r\cdot\frac{1-p}{p^{2}} -\frac{k}{\left[ p{(1+\beta)}^{2} \right]}\left[ -\left( 1-p \right)+\frac{\left( k+r \right)\left( 1-p \right)}{1+\beta}+\left[ r-p(k+r) \right]\frac{\beta}{1+\beta} \right]$ (4)

where $\beta=\frac{P_{r,p}\left\{ Y>k \right\}}{P_{r,p}\left\{ Y=k \right\}}$.

Since

$E_{r,p}\left\{ Y \right\}=0\cdot P_{r,p}\left\{ Y=0 \right\}+E_{r,p}\left\{ Y|0<Y<k \right\}\cdot{P_{r,p}\left\{ 0<Y<k \right\}+E}_{r,p}\left\{ Y|Y\geq k \right\}\cdot P_{r,p}\left\{ Y\geq k \right\}$,

we have

$$E_{r,p}\left\{ Y|0<Y<k \right\}=\frac{E_{r,p}\left\{ Y \right\}-E_{r,p}\left\{ Y|Y\geq k \right\}\cdot P_{r,p}\left\{ Y\geq k \right\}}{{Pr}_{r,p}\left\{ 0<Y<k \right\}}$$

$$=\frac{r\cdot\frac{1-p}{p}-\left( r\cdot\frac{1-p}{p}+\frac{k}{p\cdot(1+\beta)} \right)P_{r,p}\left\{ Y\geq k \right\}}{P_{r,p}\left\{ 0<Y<k \right\}}. (5)$$

Similarly, since

$E_{r,p}\left\{ Y^{2} \right\}=0\cdot{Pr}_{r,p}\left\{ Y^{2}=0 \right\}+E_{r,p}\left\{ Y^{2}|0<Y<k \right\}\cdot{P_{r,p}\left\{ 0<Y<k \right\}+E}_{r,p}\left\{ Y^{2}|Y\geq k \right\}\cdot P_{r,p}\left\{ Y\geq k \right\}$,

we have

$$E_{r,p}\left\{ Y^{2}|0<Y<k \right\}=\frac{E_{r,p}\left\{ Y^{2} \right\}-E_{r,p}\left\{ Y^{2}|Y\geq k \right\}\cdot P_{r,p}\left\{ Y\geq k \right\}}{P_{r,p}\left\{ 0<Y<k \right\}}.$$

From the definition, variance of a random variable $Z$ can be written as $Var\left\{ Z \right\}=E\left\{ Z^{2} \right\}-\left[ E\left\{ Z \right\} \right]^{2}$. Therefore,

$${Var}_{r,p}\left\{ Y|0<Y<k \right\}=E_{r,p}\left\{ Y^{2}|0<Y<k \right\}-\left( E_{r,p}\left\{ Y|0<Y<k \right\} \right)^{2}$$

$$=\frac{E_{r,p}\left\{ Y^{2} \right\}-E_{r,p}\left\{ Y^{2}|Y\geq k \right\}\cdot P_{r,p}\left\{ Y\geq k \right\}}{P_{r,p}\left\{ 0<Y<k \right\}}-\left( E_{r,p}\left\{ Y|0<Y<k \right\} \right)^{2}= \frac{{Var}_{r,p}\left\{ Y \right\}+ {E_{r,p}\left\{ Y \right\}}^{2}-\left[ {Var}_{r,p}\left\{ Y|Y\geq k \right\}+\left( E_{r,p}\left\{ Y|Y\geq k \right\} \right)^{2} \right]\cdot P_{r,p}\left\{ Y\geq k \right\}}{P_{r,p}\left\{ 0<Y<k \right\}}-\left( E_{r,p}\left\{ Y|0<Y<k \right\} \right)^{2}. (6)$$

Thus, for given $E_{r,p}\left\{ Y|0<Y<k \right\}$ and ${Var}_{r,p}\left\{ Y|0<Y<k \right\}$ we can solve $r$ and $p$ from equations (5) and (6). Numerical methods are used for the solutions. We assume the solution with an error $\left| E-\hat{E} \right|+\left| Std-\hat{Std} \right|<0.0005$ as acceptable. The results are further tested by simulations.

**References**

1. Geyer CJ. Lower-Truncated Poisson and Negative Binomial Distributions [Internet]. 2019 [cited 2020 Sep 20]. Available from: https://cran.r-project.org/web/packages/aster/vignettes/trunc.pdf

1. **SAS codes**

**/*******************************************************

**1. Prepare for simulation parameters**

*******************************************************/**

/************************************************

101 – read parameters

**************************************************/

%let dir = H:/PMT1253;

libname par "&dir/Data/Parameters";

libname xlout XLSX 'R:\BC Support Unit _Productivity simulation\Team Workspace\Huiying\simulation_par1.xlsx';

data par.MNpar1;

set xlout.MN;

run;

data par.Midpar1;

set xlout.Middle;

run;

libname xlout clear;

/*************************************************

102 – parameters of truncated NB for x = 0, equal scale

**************************************************/

%let b = 60;

data base1;

do i = 1 to 1999;

p = i/10000;

do j = 1 to 19999;

r = j/10000;

mu = r*(1-p)/p;

var = r*(1-p)/(p*p);

p0 = pdf('NEGB', 0, p, r);

pl = cdf('NEGB', &b-1, p, r); /*p(<=k)*/

pb = pdf('NEGB', &b-1, p, r); /*p(= k)*/

pl1 = cdf('NEGB', &b, p, r); /*p(<=k+1)*/

pb1 = pdf('NEGB', &b, p, r); /*p(=k+1)*/

beta = (1-pl1)/pb1;/*p(>k+1)/p(=k+1)*/

mug = mu + &b/(p*(1+beta)); /*Geyer 2019*/

mean = (mu-(1-pl)*mug)/(pl-p0);

mu2=var+mu*mu;

varg = mug + (&b-1)*(mug-mu)+mug*mu*(1+1/r)-mug*mug; /*shonkwiler 2016*/

mu2g = varg + mug*mug;

mu2m = (mu2-(1-pl)*mu2g)/(pl-p0);

sd = sqrt(mu2m - mean*mean);

output;

end;

end;

*keep p r mean sd;

run;

%put &b;

data temp1c;

set base1;

where 19.9 < mean < 20.1 and 13 < sd < 17;

d2 = abs(mean - 20) + abs(sd-14);

d3 = abs(mean - 20) + abs(sd-16);

run;

proc sql noprint;

select min(d2) into :cd2 from temp1c;

quit;

proc sql;

create table c2 as

select r, p, mean, sd, d2 from temp1c

where d2 <= &cd2;

quit;

proc sql noprint;

select min(d3) into :cd3 from temp1c;

quit;

proc sql;

create table c3 as

select r, p, mean, sd, d3 from temp1c

where d3 <= &cd3;

quit;

data temp1t;

set base1;

where 14.9 < mean < 15.1 and 11 < sd < 15;

d2 = abs(mean - 15) + abs(sd-14);

d3 = abs(mean - 15) + abs(sd-12);

run;

proc sql noprint;

select min(d2) into :td2 from temp1t;

quit;

proc sql;

create table t2 as

select r, p, mean, sd, d2 from temp1t

where d2 <= &td2 + 0.00001;

quit;

proc sql noprint;

select min(d3) into :td3 from temp1t;

quit;

%put &td3;

proc sql;

create table t3 as

select r, p, mean, sd, d3 from temp1t

where d3 <= &td3 + 0.00001;

quit;

/*******************************/

data temp2t;

set base1;

d = abs(mean - 15);

if abs(mean - 15) <= 0.0005;

rename r = r1 mean = mean1 sd = sd1;

run;

proc sort data = temp2t;

by p d;

run;

data temp2t1;

set temp2t;

by p d;

if first.p;

drop d;

run;

data temp2c;

set base1;

d = abs(mean - 20);

if abs(mean - 20) <= 0.0005;

rename r = r2 mean = mean2 sd = sd2;

run;

proc sort data = temp2c;

by p d;

run;

data temp2c1;

set temp2c;

by p d;

if first.p;

drop d;

run;

data temp3;

merge temp2c1 (in = my) temp2t1 (in = yours);

by p;

if my and yours;

d = abs(mean1-15)+ abs(mean2-20);

d1 = sd2-sd1;

sd = sqrt((sd1**2+sd2**2)/2);

d2 = abs(sd - 14) + d;

d3 = abs(mean1/sd1 - mean2/sd2);

run;

proc sort data = temp3;

by d2;

run;

data temp4;

set temp3;

if _N_ = 1;

keep p r1 r2 mean1 mean2 sd1 sd2 d2;

run;

data par.tnbpar01_c;

set c2 (in = a keep = r p d2 mean sd rename = (d2 = error))

c3 (in = b keep = r p d3 mean sd rename = (d3 = error));

if a then midpar_id = 2;

if b then midpar_id = 3;

cmean = round(mean, 1);

csd = round(sd,1);

drop mean sd;

run;

data par.tnbpar01_t;

set t2 (in = a keep = r p d2 mean sd rename = (d2 = error))

t3 (in = b keep = r p d3 mean sd rename = (d3 = error));

if a then midpar_id = 2;

if b then midpar_id = 3;

tmean = round(mean, 1);

tsd = round(sd,1);

drop mean sd;

run;

data par.tnbpar01_c;

set par.tnbpar01_c

temp4 (keep = p r2 d2 sd2

rename = (r2 = r d2 = error sd2 = csd) in = my);

if my then do;

midpar_id = 6;

cmean = 20;

end;

max = 60;

run;

data par.tnbpar01_t;

set par.tnbpar01_t

temp4 (keep = p r1 d2 sd1

rename = (r1 = r d2 = error sd1 = tsd) in = my);

if my then do;

midpar_id = 6;

tmean = 15;

end;

max = 60;

run;

/*************************************************

103 – parameters of truncated NB for x = 0, use a macro for unequal scale

**************************************************/

%macro tnbpar(mean, sd, b, dataout);

%let r0 = 1; /*may need change the values if not converge*/

%let p0 = 0.01; /*may need change the values if not converge*/

%let x = 10;

%let k = 1;

%do %while (&x > 0.001);

data temp1;

do i = -10 to 10;

p = &p0 + i*10**(-&k);

do j = -100 to 100;

r = &r0 + j*10*10**(-&k);

if r> 0 and 0 < p < 1 then do;

mu = r*(1-p)/p;

var = r*(1-p)/(p*p);

p0 = pdf('NEGB', 0, p, r);

pl = cdf('NEGB', &b-1, p, r);

pb = pdf('NEGB', &b-1, p, r);

pl1 = cdf('NEGB', &b, p, r);

pb1 = pdf('NEGB', &b, p, r);

beta = (1-pl1)/pb1;

mug = mu + &b/(p*(1+beta)); /*Geyer 2019*/

mean = (mu-(1-pl)*mug)/(pl-p0);

mu2=var+mu*mu;

varg = mug + (&b-1)*(mug-mu)+mug*mu*(1+1/r)-mug*mug; /*Shonkwiler 2016)*/

mu2g = varg + mug*mug;

mu2m = (mu2-(1-pl)*mu2g)/(pl-p0);

sd = sqrt(mu2m - mean*mean);

output;

end;

end;

end;

if p0 ne . and sd ne .;

run;

data temp1;

set temp1;

x = abs(mean - &mean) + abs(sd - &sd);

run;

proc sort data = temp1;

where x ne .;

by x;

run;

data _NUll_;

set temp1;

by x;

if _N_ = 1;

call symput("p0", p);

call symput("r0", r);

call symput("x", x);

run;

%let k = %sysevalf(&k+1);

%put &p0 &r0 &x &k;

%end;

data &dataout;

set temp1;

by x;

if _N_ = 1;

drop i j;

run;

%mend tnbpar;

%macro tnbpar_data(mid_par, midid, dataout);

data &dataout._t;

set _null_;

run;

data &dataout._c;

set _null_;

run;

proc datasets nolist;

delete tempt1-tempt2 tempc1-tempc2 par1;

quit;

data par1;

set &mid_par;

where midpar_id = &midid;

call symput("tmean", tmean);

call symput("tsd", tsd);

call symput("cmean", cmean);

call symput("csd", csd);

call symput("max", max);

run;

%tnbpar(&tmean, &tsd, &max, tempt1);

%tnbpar(&cmean, &csd, &max, tempc1);

data &dataout._t;

merge par1 tempt1;

rename x = error;

keep midpar_id tmean tsd max p r x parx;

run;

data &dataout._c;

merge par1 tempc1;

rename x = error;

keep midpar_id cmean csd max p r x parx;

run;

%mend tNBpar_data;

%tnbpar_data(par.midpar1, 1,yyy1);

%tnbpar_data(par.midpar1, 2,yyy2);

data yyy3_t;

set par.tnbpar01_t;

where midpar_id = 6;

midpar_id = 3;

parx = 0.15;

run;

data yyy3_c;

set par.tnbpar01_c;

where midpar_id = 6;

midpar_id = 3;

parx = 0.15;

run;

data par.TNBpar1_c;

set yyy1_c yyy2_c yyy3_c;

run;

data par.TNBpar1_t;

set yyy1_t yyy2_t yyy3_t;

run;

data par.tnbpar1_bl;

set par.TNBpar1_c;

where midpar_id = 2;

rename r = rbl p = pbl cmean = blmean csd = blsd;

keep cmean csd r p;

run;

/*********************************************************

104– parameters truncated Negative Binomial with covariate

*********************************************************/

%macro TNBparx(pardata, midid, treat, xpar);

proc datasets nolist;

delete temp1- temp5;

quit;

/*

proc sql noprint;

select max into :max from &pardata._&treat where midpar_id = &midid;

quit;

%put &max;

*/

data temp1;

set &pardata._&treat;

where midpar_id = &midid;

do i = 0 to 15; /*15 can be adjusted*/

do j = 0 to 9999;

d = i/10 + j/100000;

output;

end;

end;

run;

data temp2;

set temp1;

r = r + d;

mu = r*(1-p)/p;

var = r*(1-p)/(p*p);

p0 = pdf('NEGB', 0, p, r);

pl = cdf('NEGB', max-1, p, r);

pb = pdf('NEGB', max-1, p, r);

pl1 = cdf('NEGB', max, p, r);

pb1 = pdf('NEGB', max, p, r);

beta = (1-pl1)/pb1;

mug = mu + max/(p*(1+beta));/*Geyer 2019*/

mean = (mu-(1-pl)*mug)/(pl-p0);

meanx = round(mean,0.001);

diff = abs(mean-meanx);

keep r p mean diff meanx;

run;

proc sort data = temp2;

by meanx diff;

run;

data temp3;

set temp2;

by meanx diff;

if first.meanx;

run;

data temp4;

set &pardata._&treat;

where midpar_id = &midid;

do x = 0 to (max - 1);

z = &treat.mean + x*parx;

meanx = round(z, 0.001);

output;

end;

keep x meanx;

run;

data par.mid&midid.TNB_x&treat;

merge temp4 (in = my) temp3;

by meanx;

if my;

keep r p x;

run;

%mend;

%TNBparx(par.tnbpar1, 2, c);

%TNBparx(par.tnbpar1, 3, c);

%TNBparx(par.tnbpar1, 2, t);

%TNBparx(par.tnbpar1, 3, t);

**/******************************************************************

**2. Simulation**

******************************************************************/**

/***************************************************

201-Set files to keep seeds

*************************************************/

data sim.seed_x;

retain seed 549087; /*set initial seed*/

call ranuni(seed,x);

No = 1;

drop x;

run;

/*Record will be added after each run of simulation*/

data sim.record_x;

retain No 0 Seed 549087 Type MNpar_id MIDpar_id nobs start end dataset date time;

format No seed mnpar_id midpar_id nobs start end best12. date date9. time tod8.;

length Type $10

dataset $100;

run;

/**Bootstrap****/

data sim.BTseed;

retain seed 690762; /*set initial seed*/

call ranuni(seed,x);

No = 1;

drop x;

run;

data sim.BTrecord;

retain No 0 Seed 690762 Base MNpar_id MIDpar_id nobs start end dataset date time;

format No seed mnpar_id midpar_id nobs start end best12. date date9. time tod8.;

length Base $20 dataset $100;

run;

/*************************************************

202 – macro functions for simulation

**************************************************/

%macro tnb_sim_x(seed, mnpar, mnid, midpar, midid, blpar, nobs, start, end, dataout);

/*1. set look-up table*/

proc datasets nolist;

delete cfmt tfmt;

quit;

proc catalog catalog=work.formats;

delete tx (et=format) cx (et=format);

quit;

proc SQL ;

create table cfmt as

select x as start, r as label, 'cx'

as fmtname from par.mid&midid.tnb_xc;

quit ;

proc SQL ;

create table tfmt as

select x as start, r as label, 'tx'

as fmtname from par.mid&midid.tnb_xt;

quit ;

proc format cntlin = cfmt;

quit ;

proc format cntlin = tfmt;

quit ;

/*2. In put parameters parameters**/

proc datasets nolist;

delete mnpar midpar par;

quit;

data mnpar;

set par.&mnpar;

where mnpar_id = &mnid;

a1 = log(tpzero/tpmax);

a2 = log(tpmid/tpmax);

b1 = log(cpzero/cpmax) - a1;

b2 = log(cpmid/cpmax) - a2;

run;

data midpar;

merge par.&midpar._c (keep = midpar_id p max

rename = (p = pc))

par.&midpar._t (keep = midpar_id p

rename = (p = pt));

by midpar_id;

where midpar_id = &midid;

run;

data par;

merge mnpar midpar par.&blpar;

run;

/*set length*/

data _null_;

y = length(compress(&nobs, ' '));

z = max(3, z);

call symput('lnobs', compress(z,' '));

run;

data _null_;

y = length(compress(max, ' '));

z = max(3, z);

call symput('lxy', compress(z,' '));

run;

/*simulate x, get proportions, and look-up r for simulated x*/

proc datasets nolist;

delete temp1 save_seed;

quit;

data temp1;

length sim_id 5 id &lnobs x &lxy;

set par;

call streaminit(&seed);

pmaxbl = cdf("NEGB", max-1, pbl, rbl);

p0bl = cdf("NEGB", 0, pbl, rbl);

do sim_id = &start to &end;

do id = 1 to &nobs + &nobs;

px0 = rand('Uniform',0,1);

if px0 <= blpzero then x = 0;

else do;

rpbl = rand('uniform',p0bl,pmaxbl);

x = quantile("NEGB",rpbl, pbl,rbl);

end;

if id <= &nobs then do;

treat = '0';

p = pc;

p1 = exp(a1+b1+p1x*x)/(1+exp(a1+b1+p1x*x)+exp(a2+b2+p2x*x));

p2 = exp(a2+b2+p2x*x)/(1+exp(a1+b1+p1x*x)+exp(a2+b2+p2x*x));

end;

else do;

treat = '1';

p = pt;

p1 = exp(a1+p1x*x)/(1+exp(a1+p1x*x)+exp(a2+p2x*x));

p2 = exp(a2+p2x*x)/(1+exp(a1+p1x*x)+exp(a2+p2x*x));

end;

py = rand('Uniform',0,1);

if py <= p1 then do;

zero = '1';

prop = '1';

end;

else if py > p1 + p2 then do;

zero = '0';

prop = '3';

end;

else do;

zero = '0';

prop = '2';

end;

output;

end;

end;

keep sim_id treat id x max zero prop p;

run;

%let seed = &sysranend;

data &dataout;

length y &lxy;

set temp1;

if treat = '0' then r = put(x,cx.) + 0;

else if treat = '1' then r = put(x,tx.) + 0;

call streaminit(&seed);

pmax = cdf("NEGB", max-1, p, r);

p0 = cdf("NEGB", 0, p, r);

if prop = '1' then y = 0;

else if prop = '2' then do;

rp = rand('uniform',p0,pmax);

y = quantile("NEGB",rp, p,r);

end;

else y = max;

z = y/max;

keep sim_id y x treat id prop zero z;

run;

data saved_seed;

seed = &sysranend;

run;

%mend;

%macro sim_tnb_x(seeddata, recorddata, mnpar, mnid, midpar, midid, blpar, nobs, start, end);

libname MN "&dir\Data\XMN&mnid.OBS&nobs";

libname middir "&dir\Data\XMN&mnid.OBS&nobs\Mid&midid.TNB";

data _null_;

set &seeddata;

by No;

if last.No;

call symput('no0', compress(No,' '));

call symput('seed', compress(seed,' '));

run;

proc datasets nolist;

delete record zyx;

run;

data record;

length Type $10

dataset $100;

No = &No0;

seed = &seed;

Type = "TNB";

MNpar_id = &mnid;

MIDpar_id = &midid;

Nobs = &nobs;

Start = &start;

End = &end;

Dataset = "XMN&mnid.Obs&nobs\Mid&midid.TNB\TNB&start._&end";

Date = today();

Time = time();

run;

%tnb_sim_x(&seed, &mnpar, &mnid, &midpar, &midid,&blpar, &nobs, &start, &end, xyz);

*save data;

data middir.TNB&start._&end;

set xyz;

rename prop = p;

run;

*update seed;

data &seeddata;

set &seeddata saved_seed (in = my);

if my then No = &No0 + 1;

run;

*save records;

data &recorddata;

set &recorddata record;

run;

%mend sim_TNB_x;

/*****************************************************

203- Simulation

******************************************************/

options dlcreatedir;

%let dir = H:/PMT1253;

libname par "&dir/Data/Parameters";

libname sim "&dir/Data/SimData";

%include "&dir/Prog/Macro/tnbsim_x.sas" /nosource;

%let mnpardata = mnpar1;

%let midpardata = tnbpar1;

/***********************************/

%let nobs = 100;

%let mnid = 1;

%sim_TNB_x(sim.seed_x, sim.record_x, &mnpardata, &mnid, &midpardata, 2, tnbpar1_bl, &nobs, 1, 5000);

%sim_TNB_x(sim.seed_x, sim.record_x, &mnpardata, &mnid, &midpardata, 3, tnbpar1_bl, &nobs, 1, 5000);

/********************************************************/

%let mnid = 2;

%sim_TNB_x(sim.seed_x, sim.record_x, &mnpardata, &mnid, &midpardata, 2, tnbpar1_bl, &nobs, 1, 5000);

%sim_TNB_x(sim.seed_x, sim.record_x, &mnpardata, &mnid, &midpardata, 3, tnbpar1_bl, &nobs, 1, 5000);

/********************************************************/

%let mnid = 3;

%sim_TNB_x(sim.seed_x, sim.record_x, &mnpardata, &mnid, &midpardata, 2, tnbpar1_bl, &nobs, 1, 5000);

%sim_TNB_x(sim.seed_x, sim.record_x, &mnpardata, &mnid, &midpardata, 3, tnbpar1_bl, &nobs, 1, 5000);

/********************************************************/

%let nobs = 200;

/***************************************************/

%let mnid = 1;

%sim_TNB_x(sim.seed_x, sim.record_x, &mnpardata, &mnid, &midpardata, 2, tnbpar1_bl, &nobs, 1, 5000);

%sim_TNB_x(sim.seed_x, sim.record_x, &mnpardata, &mnid, &midpardata, 3, tnbpar1_bl, &nobs, 1, 5000);

/***********************************/

%let mnid = 2;

%sim_TNB_x(sim.seed_x, sim.record_x, &mnpardata, &mnid, &midpardata, 2, tnbpar1_bl, &nobs, 1, 5000);

%sim_TNB_x(sim.seed_x, sim.record_x, &mnpardata, &mnid, &midpardata, 3, tnbpar1_bl, &nobs, 1, 5000);

/***********************************/

%let mnid = 3;

%sim_TNB_x(sim.seed_x, sim.record_x, &mnpardata, &mnid, &midpardata, 2, tnbpar1_bl, &nobs, 1, 5000);

%sim_TNB_x(sim.seed_x, sim.record_x, &mnpardata, &mnid, &midpardata, 3, tnbpar1_bl, &nobs, 1, 5000);

/****************************************************/

%let nobs = 50;

/**************************************/

%let mnid = 1;

%sim_TNB_x(sim.seed_x, sim.record_x, &mnpardata, &mnid, &midpardata, 2, tnbpar1_bl, &nobs, 1, 5000);

%sim_TNB_x(sim.seed_x, sim.record_x, &mnpardata, &mnid, &midpardata, 3, tnbpar1_bl, &nobs, 1, 5000);

/***************************************************************/

%let mnid = 2;

%sim_TNB_x(sim.seed_x, sim.record_x, &mnpardata, &mnid, &midpardata, 2, tnbpar1_bl, &nobs, 1, 5000);

%sim_TNB_x(sim.seed_x, sim.record_x, &mnpardata, &mnid, &midpardata, 3, tnbpar1_bl, &nobs, 1, 5000);

/*********************************************************/

%let mnid = 3;

%sim_TNB_x(sim.seed_x, sim.record_x, &mnpardata, &mnid, &midpardata, 2, tnbpar1_bl, &nobs, 1, 5000);

%sim_TNB_x(sim.seed_x, sim.record_x, &mnpardata, &mnid, &midpardata, 3, tnbpar1_bl, &nobs, 1, 5000);

**/***************************************************

**3. regressions**

****************************************************/**

/**********************************************

301 – macro functions for regressions

***********************************************/

/***********

OLS

************/

%macro ols(datain, dataout);

proc delete data = temp1;

run;

proc delete data = &dataout;

run;

proc glm data = &datain PLOTS=NONE;

by sim_id;

class Treat;

model y = Treat x/SOLUTION clparm;

ods output ParameterEstimates = temp1

(drop = biased tvalue dependent

rename = (probt = pvalue));

run;

proc sql;

create table &dataout as

select sim_id, Estimate, stderr, pvalue, lowerCL, UpperCL

from temp1

where parameter = 'treat 0';

quit;

%mend ols;

/***********************

Negative Binomial

************************/

%macro NB(datain, by, dataout);

proc datasets nolist;

delete temp1-temp3;

quit;

proc delete data = &dataout;

proc delete data = &dataout._CS;

run;

proc genmod data = &datain (keep = &by Treat y x) PLOTS=NONE;

by &by;

class treat;

model y = treat x/dist = NB link = log;

ods output ParameterEstimates = temp1

ConvergenceStatus = temp2;

run;

data &dataout._CS;

set temp2;

where status ne 0;

run;

proc transpose data = temp1

out = &dataout (drop = _NAME_ rename = (x = xpar treat = treat_0));

where stderr > 0;

by &by;

var estimate;

id parameter;

run;

%mend NB;

/*******************************

Zero part of two-part models

********************************/

%macro pgt0(datain,by, dataout);

proc datasets nolist;

delete temp1 - temp2;

run;

proc delete data = &dataout;

proc delete data = &dataout._CS;

run;

proc logistic data = &datain (keep = &by Treat zero x) plots = none;

by &by;

class treat /param = ref;

model zero = treat x;

ods output ParameterEstimates = temp1

ConvergenceStatus = temp2;

run;

proc transpose data = temp1

out = &dataout (drop = _NAME_ rename = (ztreat = ztreat_0)) prefix = z;

by &by;

var estimate;

id variable;

run;

data &dataout._cs;

set temp2;

where status ne 0;

run;

%mend;

/**************************

Gamma for non-zeor part

***************************/

%macro ZG(datain, by, dataout);

proc datasets nolist;

delete temp1-temp2;

quit;

proc delete data = &dataout;

proc delete data = &dataout._CS;

run;

proc genmod data = &datain (keep = &by treat x y) PLOTS=NONE;

where y > 0;

by &by;

class Treat;

model y = Treat x/dist = Gamma link = log;

ods output ParameterEstimates = temp1

ConvergenceStatus = temp2;

run;

proc transpose data = temp1

out = &dataout (rename = (x = xpar treat = treat_0) drop = _NAME_);

where df ne 0;

by &by;

var estimate;

id parameter;

run;

data &dataout._CS;

set temp2;

where status ne 0;

run;

%mend ZG;

/***********************************

Zero-truncated Negative Binomial

*************************************/

%macro ZTNB(datain, by, dataout);

proc datasets nolist;

delete temp1-temp2;

quit;

proc delete data = &dataout;

proc delete data = &dataout._CS;

run;

proc nlmixed data = &datain (keep = &by treat x y);

where y > 0;

by &by;

log_mu = intercept + Treat_1*treat + xpar*x;

mu = exp(log_mu);

het = 1/alpha;

ll = lgamma(y+het) - lgamma(y + 1) - lgamma(het) - het*log(1+alpha*mu)

+ y*log(alpha*mu) - y*log(1+alpha*mu) - log(1 - (1 + alpha * mu)**-het);

model y ~ general(ll);

ods output ParameterEstimates = temp1

ConvergenceStatus = temp2;

run;

proc transpose data = temp1 out = &dataout (drop = _NAME_ rename = (alpha = scale));

by &by;

var estimate;

id parameter;

run;

data &dataout._CS;

set temp2;

where status ne 0;

run;

%mend;

/********************************

Multinomial

*********************************/

/*variable p is pre-defined,

1: if y = 0;

2: if 0 < y < max

3: if y = max

*/

%macro prob(datain,by, dataout);

proc datasets nolist;

delete temp1 - temp2;

run;

proc delete data = &dataout;

proc delete data = &dataout._CS;

run;

proc logistic data = &datain (keep = &by treat p x) plots = none;

by &by;

class treat /param = ref;

model p = treat x/link = glogit;

ods output ParameterEstimates = temp1

ConvergenceStatus = temp2;

run;

proc transpose data = temp1

out = &dataout (drop = _NAME_);

by &by;

var estimate;

id variable response;

run;

data &dataout._cs;

set temp2;

where status ne 0;

run;

%mend;

/******************************

Beta

*******************************/

%macro ZMB(datain, by, dataout);

proc datasets nolist;

delete temp1-temp4;

quit;

proc delete data = &dataout;

proc delete data = &dataout._CS;

run;

proc glimmix data = &datain (keep = &by treat x z);

where 0 < z < 1;

by &by;

class treat;

model z = treat x/dist = beta s cl;

ods output ParameterEstimates = temp1

ConvergenceStatus = temp2;

run;

proc transpose data = temp1

out = &dataout (drop = _NAME_ rename = (treat = treat_0 x = xpar));

where treat ne '1';

by &by;

var estimate;

id effect;

run;

data &dataout._CS;

set temp2;

where status ne 0;

run;

%mend;

/**********************************************

Run regressions

**********************************************/

%macro run_models_base(mnid, midid);

libname middir "&dir/Data/XMN&mnid.Obs&nobs/Mid&midid&input";

libname out "&dir/Data/XMN&mnid.Obs&nobs/Mid&midid&input/Output";

%ols(middir.&input&sim, out.ols_base&sim);

%NB(middir.&input&sim, sim_id, out.NB_base&sim);

%pgt0(middir.&input&sim, sim_id, out.pgt0_base&sim);

%ZG(middir.&input&sim, sim_id, out.zg_base&sim);

%ZTNB(middir.&input&sim, sim_id, out.ztnb_base&sim);

%prob(middir.&input&sim, sim_id, out.prob_base&sim)

%ZMB(middir.&input&sim, sim_id, out.zmb_base&sim);

%mend run_models_base;

/************************************************/

%let input = TNB;

%let sim = 1_5000;

/***********************************************/

%let nobs = 100;

ods listing close;

%run_models_base(1, 2);

%run_models_base(1, 3);

%run_models_base(2, 2);

%run_models_base(2, 3);

%run_models_base(3, 2);

%run_models_base(3, 3);

ods listing;

/********************************************/

%let nobs = 200;

ods listing close;

%run_models_base(1, 2);

%run_models_base(1, 3);

%run_models_base(2, 2);

%run_models_base(2, 3);

%run_models_base(3, 2);

%run_models_base(3, 3);

ods listing;

/********************************************/

%let nobs = 50;

ods listing close;

/*no three-parts*/

%run_models_base(1, 2);

%run_models_base(1, 3);

/*no three-parts*/

%run_models_base(2, 2);

%run_models_base(2, 3);

%run_models_base(3, 2);

%run_models_base(3, 3);

ods listing;

/************************************

303 – Bootstrap

************************************/

/********************************

Macro functions

*********************************/

%macro set(model);

%&model(bt3, sim_id replicate, tempx2);

proc sql noprint;

select count(*) into :xx from tempx2_CS;

quit;

%if &xx > 0 %then %do;

data &outdir..&model._BT_CS&start._&end;

set tempx2_CS (in = my);

run;

%end;

data &outdir..&model._BT&start._&end;

set tempx2;

run;

%mend;

%macro append(model);

%&model(bt3, sim_id replicate, tempx2);

proc sql noprint;

select count(*) into :xx from tempx2_CS;

quit;

%if &xx > 0 %then %do;

proc append base = &outdir..&model._BT_CS&start._&end

data = tempx2_CS force;

run;

quit;

%end;

proc append base = &outdir..&model._BT&start._&end

data = tempx2 force;

run;

quit;

%mend;

/*******************************************************************/

%macro run_models_BT(seeddata, recorddata, datain, reps, start, end, outdir);

data _null_;

set &seeddata;

by No;

if last.No;

if seed > 2147483647 then do; /*2^31*/

call streaminit(seed);

seed = rand('INTEGER',1,2147483647);

end;

call symput('seed', compress(seed,' '));

call symput('no0', compress(No,' '));

run;

data record;

length Base $20;

seed = &seed;

Base = "&input&insim";

N_replicates = &reps;

MNpar_id = &mnid;

Midpar_id = &midid;

Nobs = &nobs;

No = &no0;

Start = &start;

End = &end;

Date = today();

Time = time();

run;

data &recorddata;

set &recorddata record;

run;

proc datasets nolist library = &outdir;

delete seed_bt&start._&end

NB_bt&start._&end

pgt0_bt&start._&end

zg_bt&start._&end

ztnb_bt&start._&end

prob_bt&start._&end

zmb_bt&start._&end

NB_bt_CS&start._&end

pgt0_bt_CS&start._&end

zg_bt_CS&start._&end

ztnb_bt_CS&start._&end

prob_bt_CS&start._&end

zmb_bt_CS&start._&end;

run;

quit;

%do i = &start %to &end;

proc datasets nolist;

delete bt1 - bt3;

quit;

data bt1;

set &datain;

where sim_id = &i;

run;

data temp_seed;

sim_id = &i;

seed = &seed;

run;

proc surveyselect data=bt1

out= bt2

(drop = numberhits expectedhits samplingweight

rename = (replicate = rep))

outhits seed = &seed STRATUMSEED=NONE

method=urs samprate=1 reps=&reps noprint;

strata treat;

run;

/* seed for the next boots trapping*/

data _NULL_;

seed = &sysranend;

if seed > 2147483647 then do; /*2^31*/

call streaminit(seed);

seed = rand('INTEGER',1,2147483647);

end;

call symput('seed', compress(seed,' '));

run;

data bt3;

length replicate 4;

set bt2;

replicate = rep;

drop rep;

run;

proc sort data = bt3;

by sim_id replicate;

run;

%if &i = &start %then %do;

data &outdir..seed_BT&start._&end;

set temp_seed;

run;

%set(nb);

%set(pgt0);

%set(zg);

%set(ztnb);

%set(prob);

%set(zmb);

%end;

%else %do;

proc append base = &outdir..seed_BT&start._&end

data = temp_seed force;

run;

%append(nb);

%append(pgt0);

%append(zg);

%append(ztnb);

%append(prob);

%append(zmb);

%end;

%end;

data lastseed;

no = &no0 + 1;

seed = &seed;

run;

data &seeddata;

set &seeddata lastseed;

run;

%mend;

/******************************************

Run Bootstrap

******************************************/

options dlcreatedir nonotes nosource;

options threads CPUCOUNT=actual;

%let dir = H:/PMT1253;

libname sim "&dir/Data/SimData";

%let seeddata = sim.btseed;

%let recorddata = sim.btrecord;

%let insim = 1_5000;

%let input = TNB;

%let reps = 1000;

/**********************************************************

Run for all scenarios: nobs = 50, 100, 200; mind = 1, 2, 3; midid = 2, 3.

Modify the macro function run_models_BT according to regression models needed

**********************************************************/

%let nobs = 100;

%let mnid = 1;

%let midid = 1;

libname middir "&dir/Data/XMN&mnid.Obs&nobs/Mid&midid&input";

libname out "&dir/Data/XMN&mnid.Obs&nobs/Mid&midid&input/Output";

ods listing close;

%put %sysfunc(date(),date9.) %sysfunc(time(),tod8.);

%run_models_bt(&seeddata,&recorddata,middir.&input&insim, &reps, 1,5000,out);

%put %sysfunc(date(),date9.) %sysfunc(time(),tod8.);

ods listing ;

**/*********************************************

**4. Calculate estimates**

**********************************************/**

/*************************************

401- macro functions for base case calculation

***************************************/

%macro estimand(mnpardata, mnid, midpardata, midid, outdir);

proc datasets nolist;

delete temp1-temp4;

quit;

data temp1;

set &mnpardata;

where mnpar_id = &mnid;

run;

data temp2;

set &midpardata;

where midpar_id = &midid;

run;

data &outdir..Estimand_MN&mnid.Mid&midid;

retain tpzero tpmid tpmax tmean tsd

tmean_all tsd_all

cpzero cpmid cpmax cmean csd

cmean_all csd_all;

merge temp1 temp2;

tmean_all = tpmax*max+tpmid*tmean;

cmean_all = cpmax*max+cpmid*cmean;

tvar_all = tpzero*tmean_all**2 + tpmax*(max-tmean_all)**2

+ tpmid*tsd**2 + tpmid*(tmean_all-tmean)**2;

tsd_all = sqrt(tvar_all);

cvar_all = cpzero*(cmean_all**2) + cpmax*((max-cmean_all)**2)

+ cpmid*csd**2 + cpmid*(cmean_all-cmean)**2;

csd_all = sqrt(cvar_all);

estimand = cmean_all - tmean_all;

run;

%mend estimand;

/*******************************/

%macro base(mnid, midid);

libname middir "&dir/Data/XMN&mnid.Obs&nobs/Mid&midid&input";

libname out "&dir/Data/XMN&mnid.Obs&nobs/Mid&midid&input/Output";

libname est "&dir/Data/XMN&mnid.Obs&nobs/Mid&midid&input/Estimate";

/********************************************/

%tnb_estimand_x(&mnpar, &mnid, &tnbpar, &midid, &blpar, est);

/****OLS**********/

data est.ols;

set out.ols_base&sim;

do x = 0 to 59;

output;

end;

run;

/****NB**********/

data est.NB;

set out.NB_base&sim;

do x = 0 to 59;

estimate = exp(intercept+treat_0+xpar*x)

- exp(intercept+xpar*x);

output;

end;

keep sim_id x estimate;

run;

/*Two-parts**********/

data est.zg;

merge out.pgt0_base&sim (in = my) out.pgt0_base&sim._cs (in = yours keep = sim_id)

out.zg_base&sim;

by sim_id;

if my and not yours;

do x = 0 to 59;

pgt0c = exp(zintercept + ztreat_0 + zx*x)/(1+exp(zintercept + ztreat_0 + zx*x));

pgt0t = exp(zintercept + zx*x)/(1+exp(zintercept + zx*x));

estimate = pgt0c*exp(intercept+treat_0+xpar*x)-pgt0t*exp(intercept+xpar*x);

output;

end;

keep sim_id x estimate;

run;

/****************************************/

data est.ztnb;

merge out.pgt0_base&sim (in = my) out.pgt0_base&sim._cs (in = yours keep = sim_id)

out.ztnb_base&sim;

by sim_id;

if my;

do x = 0 to 59;

pgt0c = exp(zintercept + ztreat_0 + zx*x)/(1+exp(zintercept + ztreat_0 + zx*x));

pgt0t = exp(zintercept + zx*x)/(1+exp(zintercept + zx*x));

t = exp(intercept+treat_1+xpar*x);

c = exp(intercept+xpar*x);

cmu = c*(1-(scale*c+1)**(-1/scale))**(-1);

tmu = t*(1-(scale*t+1)**(-1/scale))**(-1);

estimate = pgt0c*cmu-pgt0t*tmu;

output;

end;

keep sim_id x estimate;

run;

data est.zmb;

merge out.prob_base&sim (in = my) out.prob_base&sim._cs (in = yours keep = sim_id)

out.zmb_base&sim;

by sim_id;

if my and not yours;

do x = 0 to 59;

p2c = exp(intercept2 + treat2 + x2*x)/

(1+exp(intercept1+treat1+x1*x)+exp(intercept2+treat2+x2*x));

p3c = 1/(1+exp(intercept1+treat1+x1*x)+exp(intercept2+treat2+x2*x));

p2t = exp(intercept2 + x2*x)/

(1+exp(intercept1+x1*x)+exp(intercept2+x2*x));

p3t = 1/(1+exp(intercept1+x1*x)+exp(intercept2+x2*x));

cmu = exp(intercept+treat_0+xpar*x)/(1+exp(intercept+treat_0+xpar*x));

tmu = exp(intercept+xpar*x)/(1+exp(intercept+xpar*x));

estimate = ((cmu*p2c+p3c)-(tmu*p2t+p3t))*60;

output;

end;

keep sim_id x estimate;

run;

%mend base;

/*********************************************

402- Calculate estimates from base data

*******************************************/

%let dir = H:/PMT1253;

libname par "&dir/Data/Parameters";

%let input = TNB;

%let sim = 1_5000;

%let mnpar = mnpar1;

%let tnbpar = tnbpar1;

%let blpar = tnbpar1_bl;

/*********************************************

Run the following for each nobs = 50, 100, 200

***********************************************/

%let nobs = 100;

%base(1,2);

%base(1,3);

%base(2,2);

%base(2,3);

%base(3,2);

%base(3,3);

/*************************************

403 – calculate estimates from Bootstrap

************************************************/

/*****************************************

Macro functions for Bootstrap

*****************************************/

%macro NB(datain, prob, x, dataout);

data &dataout;

set &datain;

x = &x;

estimate = exp(intercept+treat_0+xpar*&x)

- exp(intercept+xpar*&x);

keep sim_id x estimate;

run;

%mend;

/******************************************/

%macro ZG(datain, pgt0, x, dataout);

data &dataout;

merge &datain &pgt0;

by sim_id replicate;

x = &x;

pgt0c = exp(zintercept + ztreat_0 + zx*x)/(1+exp(zintercept + ztreat_0 + zx*x));

pgt0t = exp(zintercept + zx*x)/(1+exp(zintercept + zx*x));

estimate = pgt0c*exp(intercept+treat_0+xpar*x)-pgt0t*exp(intercept+xpar*x);

keep sim_id x estimate;

run;

%mend;

/********************************************/

%macro ZTNB(datain, pgt0, x, dataout);

data &dataout;

merge &datain &pgt0;

by sim_id replicate;

x = &x;

pgt0c = exp(zintercept + ztreat_0 + zx*x)/(1+exp(zintercept + ztreat_0 + zx*x));

pgt0t = exp(zintercept + zx*x)/(1+exp(zintercept + zx*x));

t = exp(intercept+treat_1+xpar*x);

c = exp(intercept+xpar*x);

cmu = c*(1-(scale*c+1)**(-1/scale))**(-1);

tmu = t*(1-(scale*t+1)**(-1/scale))**(-1);

estimate = pgt0c*cmu-pgt0t*tmu;

keep sim_id x estimate;

run;

%mend;

/********************************************/

%macro ZMB(datain, prob, x, dataout);

data &dataout;

merge &datain &prob (in = my);

by sim_id replicate;

if my;

x = &x;

p2c = exp(intercept2 + treat2 + x2*x)/

(1+exp(intercept1+treat1+x1*x)+exp(intercept2+treat2+x2*x));

p3c = 1/(1+exp(intercept1+treat1+x1*x)+exp(intercept2+treat2+x2*x));

p2t = exp(intercept2 + x2*x)/

(1+exp(intercept1+x1*x)+exp(intercept2+x2*x));

p3t = 1/(1+exp(intercept1+x1*x)+exp(intercept2+x2*x));

cmu = exp(intercept+treat_0+xpar*x)/(1+exp(intercept+treat_0+xpar*x));

tmu = exp(intercept+xpar*x)/(1+exp(intercept+xpar*x));

estimate = ((cmu*p2c+p3c)-(tmu*p2t+p3t))*60;

keep sim_id x estimate;

run;

%mend;

/********************************************/

%macro estimate(mnid, midid, prob, model, n_rep);

proc delete data = temp1;

run;

data temp1;

set out&mind&midid..&model._bt1_5000;

run;

libname est&mnid&midid "&dir/Data/XMN&mnid.Obs&nobs/Mid&midid&input/Estimate";

%do i = 0 %to 59;

proc datasets nolist;

delete temp2-temp7;

quit;

%&model.(temp1, &prob, &i, temp2);

proc freq data = temp2 noprint;

by sim_id x;

tables estimate/outcum nofreq out = temp3(drop = count cum_freq percent);

run;

proc sql;

create table temp4 as select sim_id, x, std(estimate) as StdErr

from temp3 group by sim_id, x;

create table temp5 as

select sim_id, min(estimate) as LowerCL

from temp3 where cum_pct > 2.5 group by sim_id;

create table temp6 as

select sim_id, max(estimate) as UpperCL

from temp3 where cum_pct <= 97.5 group by sim_id;

create table temp7 as

select temp4.sim_id as sim_id, x, StdErr, LowerCL, UpperCL

from temp4, temp5, temp6 where

temp4.sim_id = temp5.sim_id = temp6.sim_id;

quit;

%if &i = 0 %then %do;

data est&mnid&midid..BT_&model;

set temp7;

run;

%end;

%else %do;

proc append base = est&mnid&midid..BT_&model

data = temp7 force;

run;

quit;

%end;

%end;

%mend;

/**************************************************/

%macro setest(mnid, midid);

%set&mnid&midid(pgt0, 1000, pgt0&mnid&midid);

%estimate(&mnid, &midid, pgt0&mnid&midid, NB, 1000);

%estimate(&mnid, &midid, pgt0&mnid&midid, ZTNB, 1000);

%estimate(&mnid, &midid, pgt0&mnid&midid, ZG, 1000);

%set&mnid&midid(prob, 1000, prob&mnid&midid);

%estimate(&mnid, &midid, prob&mnid&midid, ZMB, 1000);

%mend;

/**************************************

Calculate estimates from bootstrap

***************************************/

options nonotes nosource;

options threads CPUCOUNT=actual;

%let dir = H:/PMT1253;

%let input = TNB;

%let sim = 1_5000;

/*****************************************

Run the followings for nobs = 50, 100, 200

******************************************/

%let nobs = 100;

%setest(1,2);

%setest(1,3);

%setest(2,2);

%setest(2,3);

%setest(3,2);

%setest(3,3);

**/*****************************************************

**5. Performance**

*****************************************************/**

/******************************

501-Macro functions

************************************/

%macro estdata(mnid, midid, x, model);

libname est&mnid&midid "&dir/Data/XMN&mnid.Obs&nobs/Mid&midid&input/Estimate";

data _NULL_;

set est&mnid&midid..Estimand;

where x = &x;

call symput('est', estimand);

run;

proc sql noprint;

select mean(estimate) into :mubar from est&mnid&midid..&model

where x = &x;

quit;

data &model&mnid&midid._&x;

merge est&mnid&midid..&model est&mnid&midid..bt_&model;

by sim_id x;

where x = &x;

est = &est;

pvalue = 2*(1-cdf("Normal", abs(estimate/stderr), 0, 1));

bias = estimate - &est;

sqe = (estimate - &est)**2;

var = stderr**2;

coverage = (lowerCL <= &est <= upperCL);

BE_coverage = (lowerCL <= &mubar <= upperCL);

reject = (pvalue <= 0.05);

z = 2*(1-cdf("Normal", abs((estimate-&est)/stderr), 0, 1));

run;

%mend;

/********************************************************/

%macro olsdata(mnid, midid, x, model);

libname est&mnid&midid "&dir/Data/XMN&mnid.Obs&nobs/Mid&midid&input/Estimate";

data _NULL_;

set est&mnid&midid..Estimand;

where x = &x;

call symput('est', estimand);

run;

proc sql noprint;

select mean(estimate) into :mubar from est&mnid&midid..&model

where x = &x;

quit;

data &model&mnid&midid._&x;

set est&mnid&midid..&model;

where x = &x;

est = &est;

pvalue = 2*(1-cdf("Normal", abs(estimate/stderr), 0, 1));

bias = estimate - &est;

sqe = (estimate - &est)**2;

var = stderr**2;

coverage = (lowerCL <= &est <= upperCL);

BE_coverage = (lowerCL <= &mubar <= upperCL);

reject = (pvalue <= 0.05);

z = 2*(1-cdf("Normal", abs((estimate-&est)/stderr), 0, 1));

run;

%mend;

/***************************************************/

%macro perform(model, mnid, midid, x, name);

proc datasets nolist;

delete temp1-temp8;

quit;

data temp1;

set &model&mnid&midid._&x;

sqerror = (estimate - est)**2;

var = stderr**2;

run;

proc means data = temp1 noprint;

var estimate sqerror var;

output out = temp2 (drop = _type_ _freq_)

mean = meanbar MSE modse2

std = EmpSE MSEsd modse2sd;

run;

proc means data = temp1 noprint;

var est coverage BE_coverage reject;

output out = temp3 (drop = _type_ _freq_)

mean = est coverage be_coverage power;

run;

data temp4;

retain Bias bias_mcse

EmpSE EmpSE_mcse

MSE MSE_mcse

ModSE ModSE_mcse

RE_ModSE RE_ModSE_mcse

Coverage Coverage_mcse

BE_coverage BE_coverage_mcse

Power Power_mcse;

merge temp2 temp3;

bias = meanbar - est;

bias_mcse = empse/sqrt(&nsim);

EmpSE_mcse = EmpSE/sqrt(2*(&nsim-1));

MSE_mcse = MSEsd/sqrt(&nsim);

ModSE = sqrt(modse2);

ModSE_mcse = modse2sd/(2*ModSE*sqrt(&nsim));

RE_ModSE = 100*(ModSE/EmpSE - 1);

RE_ModSE_mcse =

100*(ModSE/EmpSE)*sqrt(modse2sd**2/(4*&nsim*ModSE**4)+1/(2*(&nsim-1)));

Coverage_mcse = sqrt(Coverage*(1-Coverage)/&nsim);

BE_coverage_mcse = sqrt(BE_Coverage*(1-BE_Coverage)/&nsim);

Power_mcse = sqrt(power*(1-power)/&nsim);

run;

data temp5;

length measure1-measure8 $20.;

set temp4;

array x Coverage

BE_coverage;

do over x;

x = x*100;

end;

format Bias bias_mcse

EmpSE EmpSE_mcse

MSE MSE_mcse

ModSE ModSE_mcse

Coverage_mcse

BE_coverage_mcse 10.3

RE_ModSE RE_ModSE_mcse

Coverage

BE_coverage 10.1

Power Power_mcse 10.3;

array a Bias

Coverage

BE_coverage

EmpSE

ModSE

RE_ModSE

Power

MSE;

array b bias_mcse

Coverage_mcse

BE_coverage_mcse

EmpSE_mcse

ModSE_mcse

RE_ModSE_mcse

Power_mcse

MSE_mcse;

array c $ x1-x8;

array d $ y1-y8;

array e $ measure1-measure8;

do i = 1 to 8;

c{i} = compress(putn(a{i}, vformat(a{i})), ' ');

d{i} = compress(putn(b{i}, vformat(b{i})), ' ');

if i = 2 or i = 3 or i = 6 then do;

e{i} = catt(c{i},'%', ' (',d{i},')');

end;

else do;

e{i} = catt(c{i}, ' (',d{i},')');

end;

end;

keep measure1-measure8;

run;

proc transpose data = temp5 out = temp6 (rename = (col1 = &name));

var measure1-measure8;

run;

data per_&model&mnid&midid._&x;

set temp6;

Measure = compress(_NAME_,'measure ')+0;

drop _NAME_;

run;

%mend;

/*************************************************/

/*Need to select the models based on what are included

For example, zmb is not run for mnid = 1 and obs = 50*/

%macro pertable(mnid, midid, x);

%olsdata(&mnid, &midid, &x, ols);

%perform(ols,&mnid,&midid,&x,OLS);

%estdata(&mnid, &midid, &x,nb);

%perform(nb,&mnid,&midid,&x,NB);

%estdata(&mnid, &midid, &x,ztnb);

%perform(ztnb,&mnid,&midid,&x,TNB);

%estdata(&mnid, &midid, &x,zg);

%perform(zg,&mnid,&midid,&x,GAMMA);

%estdata(&mnid, &midid, &x,zmb);

%perform(zmb,&mnid,&midid,&x,BETA);

data per_&mnid&midid._&x;

retain perform;

merge per_OLS&mnid&midid._&x

per_NB&mnid&midid._&x

per_ZTNB&mnid&midid._&x

per_zg&mnid&midid._&x

per_zmb&mnid&midid._&x;

by measure;

mnid = &mnid;

midid = &midid;

x = &x;

format measure msr. mnid mn. midid mid.;

perform = putn(measure, vformat(measure));

weight = putn(mnid, vformat(mnid));

var_type = putn(midid, vformat(midid));

run;

%mend;

/*******************************************

402-Calculate performances

*************************************************/

option nodate nonumber;

options notes source;

%let dir = H:/PMT1253;

libname par "&dir/Data/Parameters";

libname sim "&dir/Data/SimData";

%let nsim = 5000;

%let input= tnb;

/********************************************/

proc format;

value msr 1 = 'Bias'

2 = 'Coverage'

3 = 'Bias-eliminated coverage'

4 = 'Empirical SE'

5 = 'Model SE'

6 = 'Relative error in model SE'

7 = 'Power'

8 = 'MSE';

value mn 1 = '80:15:5/60:30:10'

2 = '60:35:5/40:50:10'

3 = '50:40:10/30:55:15';

value mid 1 = 'Equal Variance'

2 = 'Unequal Scale'

3 = 'Equal Scale';

run;

/*******************************************

Run nobs = 50, 100, 200 for the followings

For nobs = 50, need to deleted the models which are not run from the macro pertable

******************************************/

%let nobs = 100;

%pertable(1,2,14);

%pertable(1,3,14);

%pertable(2,2,14);

%pertable(2,3,14);

%pertable(3,2,14);

%pertable(3,3,14);

%pertable(1,2,0);

%pertable(1,3,0);

%pertable(2,2,0);

%pertable(2,3,0);

%pertable(3,2,0);

%pertable(3,3,0);

%pertable(1,2,30);

%pertable(1,3,30);

%pertable(2,2,30);

%pertable(2,3,30);

%pertable(3,2,30);

%pertable(3,3,30);

data tempout14;

retain perform weight var_type perform OLS NB TNB GAMMA BETA;

set per_13_14 per_12_14

per_23_14 per_22_14

per_33_14 per_32_14;

if measure in (3, 6) then delete;

keep weight var_type perform OLS NB TNB GAMMA BETA measure;

rename Tnb = ZTNB gamma = ZG beta = three_part;

run;

proc sort;

by measure;

run;

data tempout0;

retain perform weight var_type OLS NB TNB GAMMA BETA;

set per_13_0 per_12_0

per_23_0 per_22_0

per_33_0 per_32_0;

if measure in (3, 6) then delete;

keep weight var_type perform OLS NB TNB GAMMA BETA measure;

rename Tnb = ZTNB gamma = ZG beta = three_part;

run;

proc sort;

by measure;

run;

data tempout30;

retain perform weight var_type OLS NB TNB GAMMA BETA;

set per_13_30 per_12_30

per_23_30 per_22_30

per_33_30 per_32_30;

if measure in (3, 6) then delete;

keep weight var_type perform OLS NB TNB GAMMA BETA measure;

rename Tnb = ZTNB gamma = ZG beta = three_part;

run;

proc sort;

by measure;

run;

libname xlout XLSX 'R:\BC Support Unit _Productivity simulation\Team Workspace\Huiying\performance_n100.xlsx';

data xlout.x_mean (rename = (perform = Measure));

set tempout14;

drop measure;

run;

data xlout.x_0 (rename = (perform = Measure));

set tempout0;

drop measure;

run;

data xlout.x_30 (rename = (perform = Measure));

set tempout30;

drop measure;

run;

libname xlout clear;

/************************************************************/
