# Supplementary material for: How to analyze work productivity loss due to health problems in randomized controlled trials? A simulation study
Source: BMC Med Res Methodol. 2021 Jun 24;21:130. doi: 10.1186/s12874-021-01330-w (PMC8223308; doi:10.1186/s12874-021-01330-w)
Supplement: Supplementary file 2 — Additional file 2: Supplementary Figure S1. Mean bias for the number of observations in each arm = 50. Supplementary Figure S2. Mean bias for the number of observations in each arm = 200. Supplementary Figure S3. Mean bias for the number of observations in each arm = 1000. Supplementary Figure S4. Mean bias for the number of observations in each arm = 2000. [file 12874_2021_1330_MOESM2_ESM.pdf]

Supplementary Figure S1. Mean bias for the number of observations in each arm = 50

Supplementary Figure S2. Mean bias for the number of observations in each arm = 200

Supplementary Figure S3. Mean bias for the number of observations in each arm = 1000

Supplementary Figure S4. Mean bias for the number of observations in each arm = 2000

Legends: OLS: ordinary least squares; NB: negative binomial; ZTNB: two-part model – logistic regression for the probability of being zero, and generalized linear regression with zero-truncated NB distribution for the non-zeros; ZG: two-part model – logistic regression for the probability of being zero, and generalized linear regression with Gamma distribution for the non-zeros; Three-part: multinomial logistic regression for the probabilities of being zero and 60 and generalized linear regression with Beta distribution for the those with values in (0, 60) (transformed to (0, 1)).

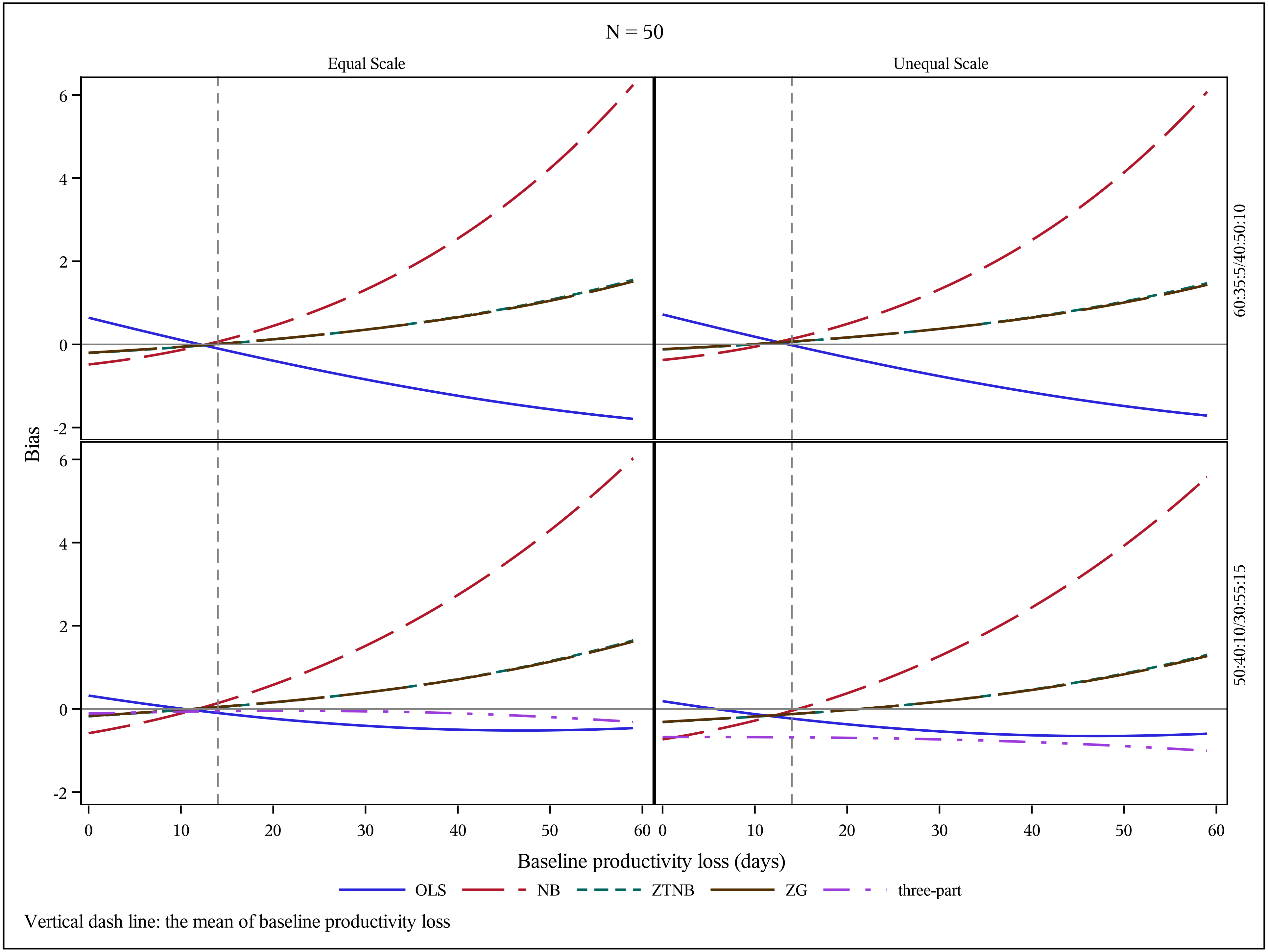

N = 50
Equal Scale
Unequal Scale
60:35:5/40:50:10
50:40:10/30:55:15
Bias
6
4
2
0
-2
0
10
20
30
40
50
60
Baseline productivity loss (days)
OLS
NB
ZTNB
ZG
three-part
Vertical dash line: the mean of baseline productivity loss

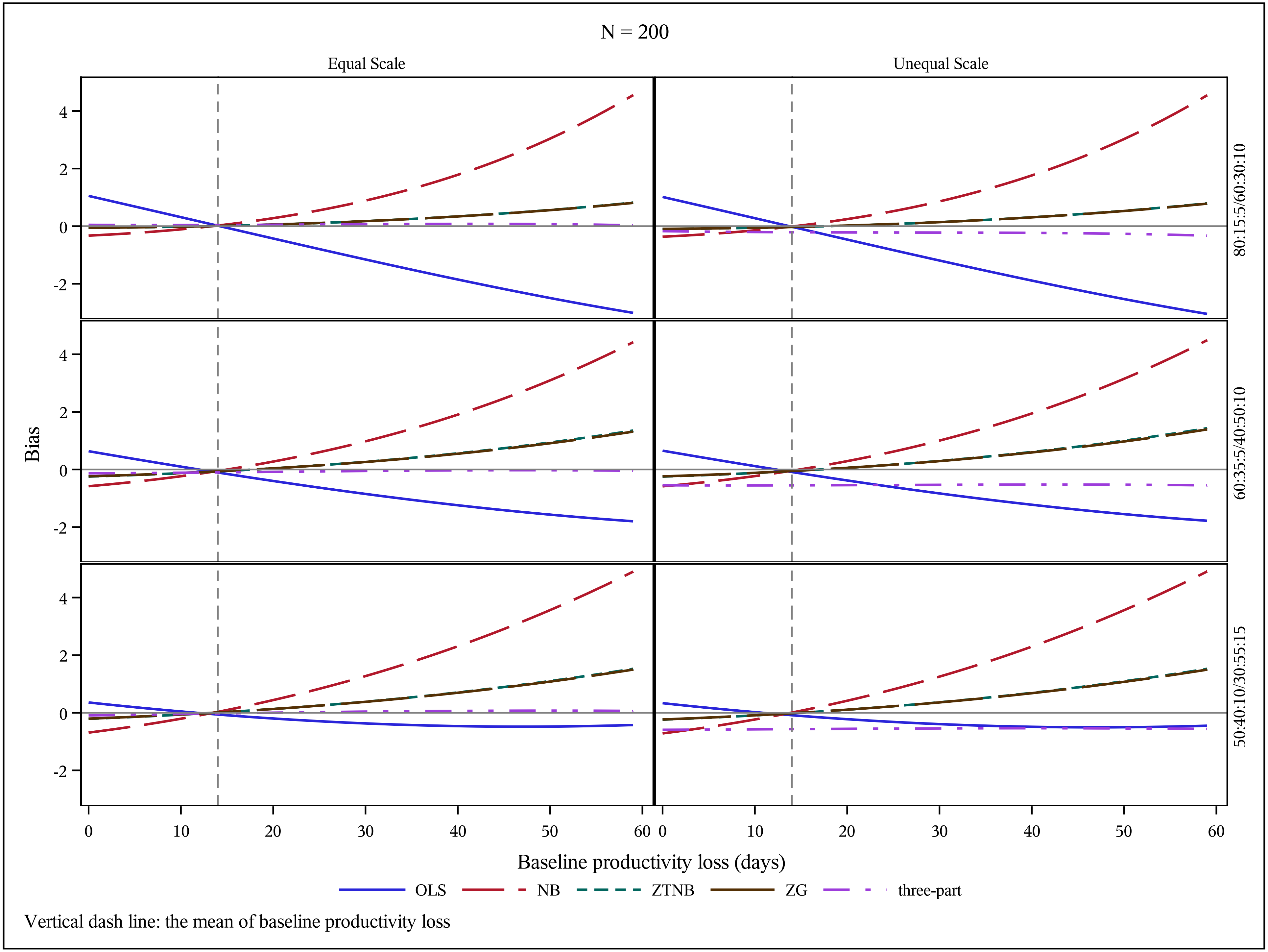

N = 200
Equal Scale
Unequal Scale
Bias
80:15:5/60:30:10
60:35:5/40:50:10
50:40:10/30:55:15
4
2
0
-2
0
10
20
30
40
50
60
Baseline productivity loss (days)
OLS
NB
ZTNB
ZG
three-part
Vertical dash line: the mean of baseline productivity loss

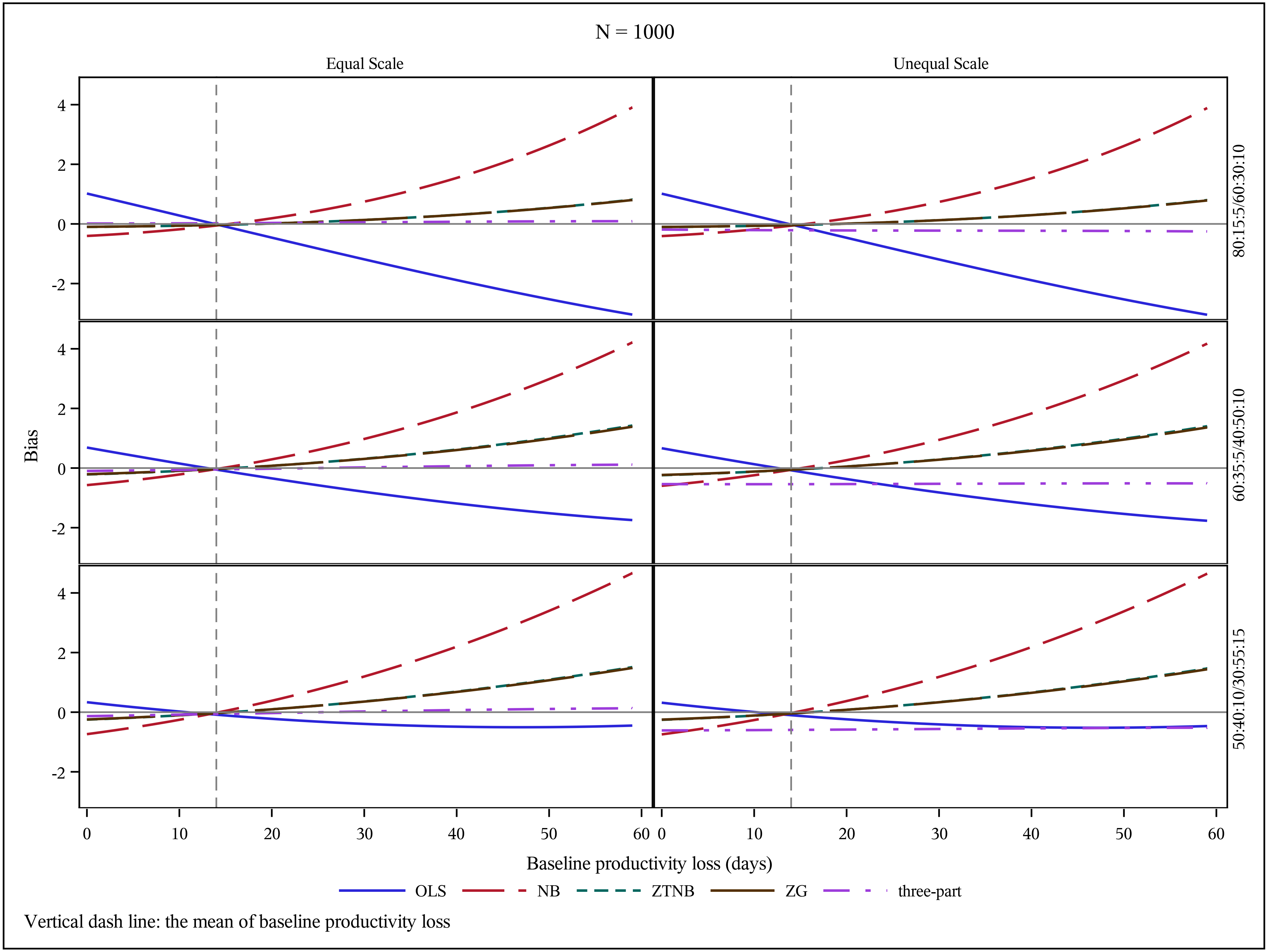

N = 1000
Equal Scale
Unequal Scale
80:15:5/60:30:10
60:35:5/40:50:10
50:40:10/30:55:15
Bias
4
2
0
-2
0
10
20
30
40
50
60
Baseline productivity loss (days)
OLS
NB
ZTNB
ZG
three-part
Vertical dash line: the mean of baseline productivity loss

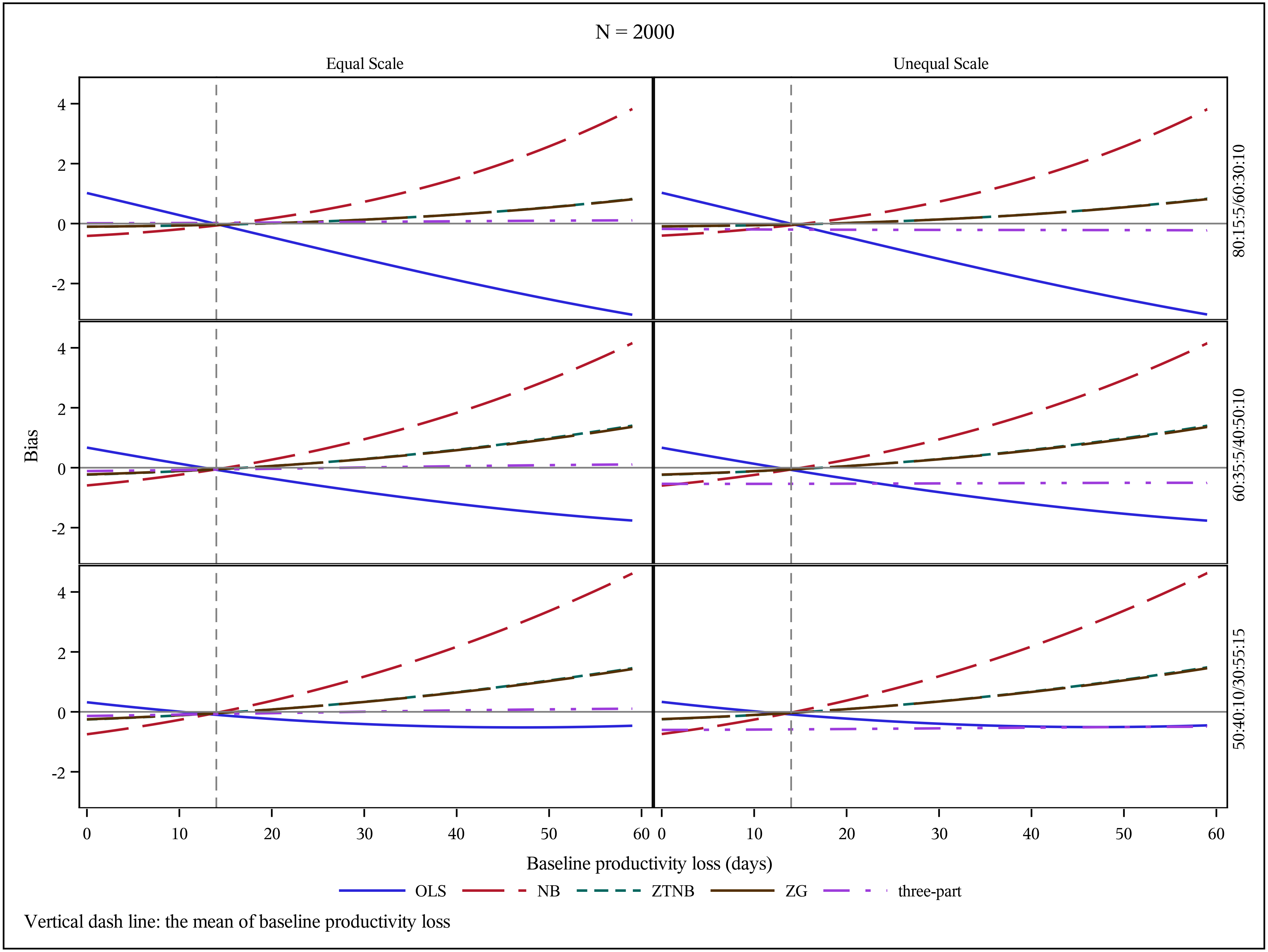

N = 2000
Equal Scale
Unequal Scale
80:15:5/60:30:10
60:35:5/40:50:10
50:40:10/30:55:15
Bias
4
2
0
-2
0
10
20
30
40
50
60
Baseline productivity loss (days)
OLS
NB
ZTNB
ZG
three-part
Vertical dash line: the mean of baseline productivity loss
