# Supplementary material for: How to analyze work productivity loss due to health problems in randomized controlled trials? A simulation study
Source: BMC Med Res Methodol. 2021 Jun 24;21:130. doi: 10.1186/s12874-021-01330-w (PMC8223308; doi:10.1186/s12874-021-01330-w)
Supplement: Supplementary file 3 — Additional file 3: Supplementary Table S1. Performance measures for the number of observations each arm = 50. Supplementary Table S2. Performance measures for the number of observations each arm = 100. Supplementary Table S3. Performance measures for the number of observations each arm = 200. [file 12874_2021_1330_MOESM3_ESM.docx]

**Supplementary Table S1. Performance measures for the number of observations each arm = 50.**

| **Measure** | **Distribution of productivity loss outcome** | **Scales in the two arms^a^** | **OLS** | **NB** | **ZTNB** | **ZG** | **Three-part** |
| --- | --- | --- | --- | --- | --- | --- | --- |
| $\boldsymbol{x}$ **= mean** |  |  |  |  |  |  |  |
| Bias | 60:35:5/40:50:10 | Equal Scale | -0.098 (0.055) | 0.066 (0.057) | 0.004 (0.056) | 0.010 (0.056) |  |
| Bias | 60:35:5/40:50:10 | Unequal Scale | -0.020 (0.055) | 0.133 (0.058) | 0.062 (0.056) | 0.068 (0.056) |  |
| Bias | 50:40:10/30:55:15 | Equal Scale | -0.098 (0.062) | 0.139 (0.064) | 0.041 (0.063) | 0.046 (0.063) | -0.056 (0.061) |
| Bias | 50:40:10/30:55:15 | Unequal Scale | -0.232 (0.062) | -0.045 (0.064) | -0.127 (0.063) | -0.123 (0.063) | -0.683 (0.062) |
| Coverage | 60:35:5/40:50:10 | Equal Scale | 95.2% (0.003) | 94.4% (0.003) | 94.2% (0.003) | 94.1% (0.003) |  |
| Coverage | 60:35:5/40:50:10 | Unequal Scale | 95.2% (0.003) | 94.2% (0.003) | 94.3% (0.003) | 94.3% (0.003) |  |
| Coverage | 50:40:10/30:55:15 | Equal Scale | 94.5% (0.003) | 94.0% (0.003) | 94.0% (0.003) | 94.0% (0.003) | 94.1% (0.003) |
| Coverage | 50:40:10/30:55:15 | Unequal Scale | 94.8% (0.003) | 94.3% (0.003) | 94.4% (0.003) | 94.4% (0.003) | 94.3% (0.003) |
| Empirical SE | 60:35:5/40:50:10 | Equal Scale | 3.883 (0.039) | 4.053 (0.041) | 3.957 (0.040) | 3.962 (0.040) |  |
| Empirical SE | 60:35:5/40:50:10 | Unequal Scale | 3.895 (0.039) | 4.070 (0.041) | 3.965 (0.040) | 3.970 (0.040) |  |
| Empirical SE | 50:40:10/30:55:15 | Equal Scale | 4.383 (0.044) | 4.558 (0.046) | 4.466 (0.045) | 4.471 (0.045) | 4.335 (0.043) |
| Empirical SE | 50:40:10/30:55:15 | Unequal Scale | 4.381 (0.044) | 4.542 (0.045) | 4.468 (0.045) | 4.472 (0.045) | 4.353 (0.044) |
| Model SE | 60:35:5/40:50:10 | Equal Scale | 3.887 (0.004) | 4.126 (0.007) | 3.948 (0.005) | 3.954 (0.005) |  |
| Model SE | 60:35:5/40:50:10 | Unequal Scale | 3.908 (0.004) | 4.137 (0.006) | 3.967 (0.005) | 3.972 (0.005) |  |
| Model SE | 50:40:10/30:55:15 | Equal Scale | 4.331 (0.004) | 4.556 (0.006) | 4.416 (0.005) | 4.423 (0.005) | 4.266 (0.005) |
| Model SE | 50:40:10/30:55:15 | Unequal Scale | 4.348 (0.004) | 4.558 (0.005) | 4.431 (0.005) | 4.436 (0.005) | 4.289 (0.005) |
| MSE | 60:35:5/40:50:10 | Equal Scale | 15.085 (0.298) | 16.430 (0.332) | 15.656 (0.308) | 15.694 (0.309) |  |
| MSE | 60:35:5/40:50:10 | Unequal Scale | 15.168 (0.305) | 16.579 (0.349) | 15.722 (0.319) | 15.759 (0.320) |  |
| MSE | 50:40:10/30:55:15 | Equal Scale | 19.218 (0.383) | 20.786 (0.424) | 19.944 (0.398) | 19.989 (0.399) | 18.791 (0.375) |
| MSE | 50:40:10/30:55:15 | Unequal Scale | 19.243 (0.383) | 20.624 (0.411) | 19.975 (0.396) | 20.009 (0.397) | 19.416 (0.390) |
| Power | 60:35:5/40:50:10 | Equal Scale | 0.582 (0.007) | 0.558 (0.007) | 0.586 (0.007) | 0.585 (0.007) |  |
| Power | 60:35:5/40:50:10 | Unequal Scale | 0.586 (0.007) | 0.559 (0.007) | 0.581 (0.007) | 0.581 (0.007) |  |
| Power | 50:40:10/30:55:15 | Equal Scale | 0.486 (0.007) | 0.472 (0.007) | 0.486 (0.007) | 0.485 (0.007) | 0.500 (0.007) |
| Power | 50:40:10/30:55:15 | Unequal Scale | 0.468 (0.007) | 0.456 (0.007) | 0.468 (0.007) | 0.467 (0.007) | 0.443 (0.007) |
| $\boldsymbol{x}$ **= 0** |  |  |  |  |  |  |  |
| Bias | 60:35:5/40:50:10 | Equal Scale | 0.642 (0.055) | -0.483 (0.050) | -0.208 (0.051) | -0.200 (0.051) |  |
| Bias | 60:35:5/40:50:10 | Unequal Scale | 0.721 (0.055) | -0.375 (0.051) | -0.121 (0.051) | -0.114 (0.051) |  |
| Bias | 50:40:10/30:55:15 | Equal Scale | 0.322 (0.062) | -0.584 (0.057) | -0.178 (0.058) | -0.172 (0.058) | -0.112 (0.058) |
| Bias | 50:40:10/30:55:15 | Unequal Scale | 0.187 (0.062) | -0.733 (0.057) | -0.318 (0.058) | -0.312 (0.058) | -0.676 (0.058) |
| Coverage | 60:35:5/40:50:10 | Equal Scale | 94.5% (0.003) | 94.2% (0.003) | 94.2% (0.003) | 94.2% (0.003) |  |
| Coverage | 60:35:5/40:50:10 | Unequal Scale | 94.8% (0.003) | 93.8% (0.003) | 94.2% (0.003) | 94.2% (0.003) |  |
| Coverage | 50:40:10/30:55:15 | Equal Scale | 94.3% (0.003) | 93.7% (0.003) | 93.9% (0.003) | 93.8% (0.003) | 94.4% (0.003) |
| Coverage | 50:40:10/30:55:15 | Unequal Scale | 94.7% (0.003) | 94.1% (0.003) | 94.5% (0.003) | 94.5% (0.003) | 94.6% (0.003) |
| Empirical SE | 60:35:5/40:50:10 | Equal Scale | 3.883 (0.039) | 3.566 (0.036) | 3.577 (0.036) | 3.581 (0.036) |  |
| Empirical SE | 60:35:5/40:50:10 | Unequal Scale | 3.895 (0.039) | 3.630 (0.036) | 3.620 (0.036) | 3.623 (0.036) |  |
| Empirical SE | 50:40:10/30:55:15 | Equal Scale | 4.383 (0.044) | 4.045 (0.040) | 4.099 (0.041) | 4.105 (0.041) | 4.102 (0.041) |
| Empirical SE | 50:40:10/30:55:15 | Unequal Scale | 4.381 (0.044) | 4.006 (0.040) | 4.088 (0.041) | 4.093 (0.041) | 4.124 (0.041) |
| Model SE | 60:35:5/40:50:10 | Equal Scale | 3.887 (0.004) | 3.761 (0.012) | 3.615 (0.007) | 3.619 (0.007) |  |
| Model SE | 60:35:5/40:50:10 | Unequal Scale | 3.908 (0.004) | 3.796 (0.013) | 3.647 (0.007) | 3.650 (0.007) |  |
| Model SE | 50:40:10/30:55:15 | Equal Scale | 4.331 (0.004) | 4.097 (0.011) | 4.075 (0.007) | 4.080 (0.007) | 4.180 (0.008) |
| Model SE | 50:40:10/30:55:15 | Unequal Scale | 4.348 (0.004) | 4.099 (0.010) | 4.100 (0.007) | 4.105 (0.007) | 4.213 (0.008) |
| MSE | 60:35:5/40:50:10 | Equal Scale | 15.488 (0.304) | 12.945 (0.273) | 12.835 (0.256) | 12.863 (0.256) |  |
| MSE | 60:35:5/40:50:10 | Unequal Scale | 15.686 (0.317) | 13.317 (0.297) | 13.114 (0.280) | 13.139 (0.280) |  |
| MSE | 50:40:10/30:55:15 | Equal Scale | 19.312 (0.385) | 16.704 (0.366) | 16.834 (0.351) | 16.874 (0.352) | 16.837 (0.349) |
| MSE | 50:40:10/30:55:15 | Unequal Scale | 19.224 (0.382) | 16.578 (0.336) | 16.806 (0.339) | 16.843 (0.339) | 17.465 (0.353) |
| Power | 60:35:5/40:50:10 | Equal Scale | 0.582 (0.007) | 0.519 (0.007) | 0.574 (0.007) | 0.574 (0.007) |  |
| Power | 60:35:5/40:50:10 | Unequal Scale | 0.586 (0.007) | 0.520 (0.007) | 0.567 (0.007) | 0.567 (0.007) |  |
| Power | 50:40:10/30:55:15 | Equal Scale | 0.486 (0.007) | 0.457 (0.007) | 0.493 (0.007) | 0.493 (0.007) | 0.479 (0.007) |
| Power | 50:40:10/30:55:15 | Unequal Scale | 0.468 (0.007) | 0.437 (0.007) | 0.472 (0.007) | 0.471 (0.007) | 0.421 (0.007) |
| $\boldsymbol{x}$ **= 30** |  |  |  |  |  |  |  |
| Bias | 60:35:5/40:50:10 | Equal Scale | -0.842 (0.055) | 1.314 (0.078) | 0.355 (0.066) | 0.354 (0.066) |  |
| Bias | 60:35:5/40:50:10 | Unequal Scale | -0.764 (0.055) | 1.322 (0.078) | 0.375 (0.066) | 0.373 (0.066) |  |
| Bias | 50:40:10/30:55:15 | Equal Scale | -0.406 (0.062) | 1.516 (0.081) | 0.394 (0.073) | 0.394 (0.073) | -0.058 (0.067) |
| Bias | 50:40:10/30:55:15 | Unequal Scale | -0.540 (0.062) | 1.270 (0.081) | 0.179 (0.073) | 0.177 (0.073) | -0.733 (0.068) |
| Coverage | 60:35:5/40:50:10 | Equal Scale | 94.6% (0.003) | 93.7% (0.003) | 94.3% (0.003) | 94.3% (0.003) |  |
| Coverage | 60:35:5/40:50:10 | Unequal Scale | 94.8% (0.003) | 93.8% (0.003) | 94.2% (0.003) | 94.2% (0.003) |  |
| Coverage | 50:40:10/30:55:15 | Equal Scale | 94.6% (0.003) | 93.5% (0.003) | 94.1% (0.003) | 94.1% (0.003) | 95.0% (0.003) |
| Coverage | 50:40:10/30:55:15 | Unequal Scale | 94.9% (0.003) | 93.7% (0.003) | 94.5% (0.003) | 94.5% (0.003) | 94.7% (0.003) |
| Empirical SE | 60:35:5/40:50:10 | Equal Scale | 3.883 (0.039) | 5.498 (0.055) | 4.681 (0.047) | 4.682 (0.047) |  |
| Empirical SE | 60:35:5/40:50:10 | Unequal Scale | 3.895 (0.039) | 5.509 (0.055) | 4.672 (0.047) | 4.673 (0.047) |  |
| Empirical SE | 50:40:10/30:55:15 | Equal Scale | 4.383 (0.044) | 5.761 (0.058) | 5.157 (0.052) | 5.157 (0.052) | 4.755 (0.048) |
| Empirical SE | 50:40:10/30:55:15 | Unequal Scale | 4.381 (0.044) | 5.717 (0.057) | 5.132 (0.051) | 5.130 (0.051) | 4.781 (0.048) |
| Model SE | 60:35:5/40:50:10 | Equal Scale | 3.887 (0.004) | 6.551 (0.137) | 4.822 (0.011) | 4.827 (0.011) |  |
| Model SE | 60:35:5/40:50:10 | Unequal Scale | 3.908 (0.004) | 6.348 (0.079) | 4.784 (0.010) | 4.786 (0.010) |  |
| Model SE | 50:40:10/30:55:15 | Equal Scale | 4.331 (0.004) | 6.258 (0.033) | 5.234 (0.010) | 5.238 (0.010) | 4.837 (0.009) |
| Model SE | 50:40:10/30:55:15 | Unequal Scale | 4.348 (0.004) | 6.160 (0.029) | 5.202 (0.009) | 5.203 (0.009) | 4.850 (0.009) |
| MSE | 60:35:5/40:50:10 | Equal Scale | 15.785 (0.314) | 31.949 (0.984) | 22.031 (0.448) | 22.041 (0.449) |  |
| MSE | 60:35:5/40:50:10 | Unequal Scale | 15.750 (0.316) | 32.094 (1.070) | 21.961 (0.462) | 21.971 (0.463) |  |
| MSE | 50:40:10/30:55:15 | Equal Scale | 19.373 (0.386) | 35.487 (0.871) | 26.746 (0.551) | 26.750 (0.552) | 22.611 (0.459) |
| MSE | 50:40:10/30:55:15 | Unequal Scale | 19.481 (0.388) | 34.296 (0.818) | 26.359 (0.542) | 26.339 (0.542) | 23.387 (0.487) |
| Power | 60:35:5/40:50:10 | Equal Scale | 0.582 (0.007) | 0.404 (0.007) | 0.524 (0.007) | 0.522 (0.007) |  |
| Power | 60:35:5/40:50:10 | Unequal Scale | 0.586 (0.007) | 0.428 (0.007) | 0.532 (0.007) | 0.531 (0.007) |  |
| Power | 50:40:10/30:55:15 | Equal Scale | 0.486 (0.007) | 0.382 (0.007) | 0.421 (0.007) | 0.421 (0.007) | 0.451 (0.007) |
| Power | 50:40:10/30:55:15 | Unequal Scale | 0.468 (0.007) | 0.376 (0.007) | 0.410 (0.007) | 0.409 (0.007) | 0.389 (0.007) |
| ^a^ for truncated negative binomial distributions of productivity loss outcomes in the two arms;  OLS: ordinary least squares; NB: negative binomial; ZTNB: two-part model – logistic regression for the probability of being zero, and generalized linear regression with zero-truncated NB distribution for the non-zeros; ZG: two-part model – logistic regression for the probability of being zero, and generalized linear regression with Gamma distribution for the non-zeros; Three-part: multinomial logistic regression for the probabilities of being zero and 60 and generalized linear regression with Beta distribution for the those with values in (0, 60) (transformed to (0, 1)). | | | | | | | |

**Supplementary Table S2. Performance measures for the number of observations each arm = 100.**

| **Measure** | **Distribution of productivity loss outcome** | **Scales in the two arms^a^** | **OLS** | **NB** | **ZTNB** | **ZG** | **Three-part** | |
| --- | --- | --- | --- | --- | --- | --- | --- | --- |
| $\boldsymbol{x}$ **= mean** |  |  |  |  |  |  |  | |
| Bias | 80:15:5/60:30:10 | Equal Scale | -0.066 (0.038) | 0.012 (0.040) | -0.060 (0.038) | -0.057 (0.038) | -0.050 (0.038) |  |
| Bias | 80:15:5/60:30:10 | Unequal Scale | -0.009 (0.039) | 0.078 (0.041) | -0.008 (0.039) | -0.005 (0.039) | -0.224 (0.038) |  |
| Bias | 60:35:5/40:50:10 | Equal Scale | -0.073 (0.039) | 0.032 (0.039) | -0.010 (0.039) | -0.005 (0.039) | -0.061 (0.038) |  |
| Bias | 60:35:5/40:50:10 | Unequal Scale | -0.085 (0.039) | -0.020 (0.040) | -0.044 (0.039) | -0.041 (0.039) | -0.536 (0.038) |  |
| Bias | 50:40:10/30:55:15 | Equal Scale | -0.137 (0.043) | -0.020 (0.044) | -0.058 (0.043) | -0.054 (0.043) | -0.110 (0.042) |  |
| Bias | 50:40:10/30:55:15 | Unequal Scale | -0.076 (0.043) | 0.039 (0.044) | -0.000 (0.044) | 0.002 (0.044) | -0.539 (0.043) |  |
| Coverage | 80:15:5/60:30:10 | Equal Scale | 95.2% (0.003) | 94.7% (0.003) | 94.5% (0.003) | 94.5% (0.003) | 94.6% (0.003) |  |
| Coverage | 80:15:5/60:30:10 | Unequal Scale | 94.7% (0.003) | 94.4% (0.003) | 94.3% (0.003) | 94.3% (0.003) | 94.4% (0.003) |  |
| Coverage | 60:35:5/40:50:10 | Equal Scale | 95.2% (0.003) | 94.7% (0.003) | 94.8% (0.003) | 94.7% (0.003) | 94.8% (0.003) |  |
| Coverage | 60:35:5/40:50:10 | Unequal Scale | 94.5% (0.003) | 94.3% (0.003) | 94.3% (0.003) | 94.3% (0.003) | 94.1% (0.003) |  |
| Coverage | 50:40:10/30:55:15 | Equal Scale | 95.2% (0.003) | 95.0% (0.003) | 94.9% (0.003) | 94.9% (0.003) | 95.2% (0.003) |  |
| Coverage | 50:40:10/30:55:15 | Unequal Scale | 95.4% (0.003) | 94.9% (0.003) | 94.8% (0.003) | 94.8% (0.003) | 94.8% (0.003) |  |
| Empirical SE | 80:15:5/60:30:10 | Equal Scale | 2.708 (0.027) | 2.813 (0.028) | 2.714 (0.027) | 2.716 (0.027) | 2.677 (0.027) |  |
| Empirical SE | 80:15:5/60:30:10 | Unequal Scale | 2.756 (0.028) | 2.867 (0.029) | 2.766 (0.028) | 2.768 (0.028) | 2.718 (0.027) |  |
| Empirical SE | 60:35:5/40:50:10 | Equal Scale | 2.734 (0.027) | 2.793 (0.028) | 2.750 (0.028) | 2.752 (0.028) | 2.671 (0.027) |  |
| Empirical SE | 60:35:5/40:50:10 | Unequal Scale | 2.744 (0.027) | 2.806 (0.028) | 2.761 (0.028) | 2.763 (0.028) | 2.689 (0.027) |  |
| Empirical SE | 50:40:10/30:55:15 | Equal Scale | 3.025 (0.030) | 3.096 (0.031) | 3.056 (0.031) | 3.058 (0.031) | 2.981 (0.030) |  |
| Empirical SE | 50:40:10/30:55:15 | Unequal Scale | 3.051 (0.031) | 3.119 (0.031) | 3.082 (0.031) | 3.084 (0.031) | 3.013 (0.030) |  |
| Model SE | 80:15:5/60:30:10 | Equal Scale | 2.703 (0.002) | 2.882 (0.004) | 2.708 (0.003) | 2.711 (0.003) | 2.652 (0.003) |  |
| Model SE | 80:15:5/60:30:10 | Unequal Scale | 2.719 (0.002) | 2.892 (0.005) | 2.726 (0.003) | 2.728 (0.003) | 2.671 (0.003) |  |
| Model SE | 60:35:5/40:50:10 | Equal Scale | 2.747 (0.002) | 2.832 (0.003) | 2.766 (0.002) | 2.769 (0.002) | 2.679 (0.002) |  |
| Model SE | 60:35:5/40:50:10 | Unequal Scale | 2.757 (0.002) | 2.839 (0.003) | 2.778 (0.002) | 2.781 (0.002) | 2.699 (0.002) |  |
| Model SE | 50:40:10/30:55:15 | Equal Scale | 3.052 (0.002) | 3.140 (0.002) | 3.087 (0.002) | 3.089 (0.002) | 3.002 (0.002) |  |
| Model SE | 50:40:10/30:55:15 | Unequal Scale | 3.064 (0.002) | 3.149 (0.002) | 3.097 (0.002) | 3.099 (0.002) | 3.019 (0.002) |  |
| MSE | 80:15:5/60:30:10 | Equal Scale | 7.337 (0.146) | 7.912 (0.163) | 7.369 (0.148) | 7.379 (0.148) | 7.168 (0.145) |  |
| MSE | 80:15:5/60:30:10 | Unequal Scale | 7.596 (0.152) | 8.221 (0.168) | 7.648 (0.153) | 7.659 (0.153) | 7.434 (0.148) |  |
| MSE | 60:35:5/40:50:10 | Equal Scale | 7.477 (0.145) | 7.800 (0.153) | 7.562 (0.147) | 7.573 (0.147) | 7.139 (0.139) |  |
| MSE | 60:35:5/40:50:10 | Unequal Scale | 7.538 (0.153) | 7.872 (0.157) | 7.625 (0.153) | 7.636 (0.153) | 7.519 (0.149) |  |
| MSE | 50:40:10/30:55:15 | Equal Scale | 9.167 (0.181) | 9.585 (0.190) | 9.341 (0.185) | 9.355 (0.185) | 8.898 (0.175) |  |
| MSE | 50:40:10/30:55:15 | Unequal Scale | 9.313 (0.182) | 9.726 (0.188) | 9.494 (0.183) | 9.507 (0.184) | 9.369 (0.183) |  |
| Power | 80:15:5/60:30:10 | Equal Scale | 0.813 (0.006) | 0.789 (0.006) | 0.815 (0.005) | 0.814 (0.005) | 0.831 (0.005) |  |
| Power | 80:15:5/60:30:10 | Unequal Scale | 0.816 (0.005) | 0.791 (0.006) | 0.811 (0.006) | 0.811 (0.006) | 0.812 (0.006) |  |
| Power | 60:35:5/40:50:10 | Equal Scale | 0.865 (0.005) | 0.861 (0.005) | 0.868 (0.005) | 0.868 (0.005) | 0.886 (0.004) |  |
| Power | 60:35:5/40:50:10 | Unequal Scale | 0.863 (0.005) | 0.852 (0.005) | 0.862 (0.005) | 0.861 (0.005) | 0.845 (0.005) |  |
| Power | 50:40:10/30:55:15 | Equal Scale | 0.771 (0.006) | 0.761 (0.006) | 0.769 (0.006) | 0.769 (0.006) | 0.789 (0.006) |  |
| Power | 50:40:10/30:55:15 | Unequal Scale | 0.770 (0.006) | 0.762 (0.006) | 0.767 (0.006) | 0.767 (0.006) | 0.733 (0.006) |  |
| $\boldsymbol{x}$ **= 0** |  |  |  |  |  |  |  | |
| Bias | 80:15:5/60:30:10 | Equal Scale | 0.969 (0.038) | -0.358 (0.035) | -0.140 (0.034) | -0.138 (0.034) | -0.034 (0.034) | |
| Bias | 80:15:5/60:30:10 | Unequal Scale | 1.026 (0.039) | -0.284 (0.035) | -0.071 (0.035) | -0.069 (0.035) | -0.156 (0.035) | |
| Bias | 60:35:5/40:50:10 | Equal Scale | 0.667 (0.039) | -0.518 (0.035) | -0.203 (0.035) | -0.195 (0.035) | -0.077 (0.035) | |
| Bias | 60:35:5/40:50:10 | Unequal Scale | 0.655 (0.039) | -0.553 (0.035) | -0.233 (0.035) | -0.226 (0.035) | -0.516 (0.035) | |
| Bias | 50:40:10/30:55:15 | Equal Scale | 0.282 (0.043) | -0.725 (0.038) | -0.273 (0.039) | -0.267 (0.039) | -0.184 (0.039) | |
| Bias | 50:40:10/30:55:15 | Unequal Scale | 0.344 (0.043) | -0.672 (0.039) | -0.208 (0.040) | -0.203 (0.040) | -0.562 (0.040) | |
| Coverage | 80:15:5/60:30:10 | Equal Scale | 93.4% (0.004) | 94.3% (0.003) | 94.7% (0.003) | 94.7% (0.003) | 95.0% (0.003) | |
| Coverage | 80:15:5/60:30:10 | Unequal Scale | 93.1% (0.004) | 93.9% (0.003) | 94.2% (0.003) | 94.2% (0.003) | 94.2% (0.003) | |
| Coverage | 60:35:5/40:50:10 | Equal Scale | 94.2% (0.003) | 94.3% (0.003) | 94.4% (0.003) | 94.4% (0.003) | 95.1% (0.003) | |
| Coverage | 60:35:5/40:50:10 | Unequal Scale | 94.3% (0.003) | 93.6% (0.003) | 94.3% (0.003) | 94.3% (0.003) | 94.4% (0.003) | |
| Coverage | 50:40:10/30:55:15 | Equal Scale | 95.2% (0.003) | 93.9% (0.003) | 94.9% (0.003) | 94.8% (0.003) | 95.1% (0.003) | |
| Coverage | 50:40:10/30:55:15 | Unequal Scale | 95.0% (0.003) | 93.9% (0.003) | 94.8% (0.003) | 94.8% (0.003) | 94.7% (0.003) | |
| Empirical SE | 80:15:5/60:30:10 | Equal Scale | 2.708 (0.027) | 2.447 (0.024) | 2.414 (0.024) | 2.416 (0.024) | 2.415 (0.024) | |
| Empirical SE | 80:15:5/60:30:10 | Unequal Scale | 2.756 (0.028) | 2.499 (0.025) | 2.465 (0.025) | 2.467 (0.025) | 2.456 (0.025) | |
| Empirical SE | 60:35:5/40:50:10 | Equal Scale | 2.734 (0.027) | 2.448 (0.024) | 2.495 (0.025) | 2.498 (0.025) | 2.504 (0.025) | |
| Empirical SE | 60:35:5/40:50:10 | Unequal Scale | 2.744 (0.027) | 2.458 (0.025) | 2.489 (0.025) | 2.492 (0.025) | 2.497 (0.025) | |
| Empirical SE | 50:40:10/30:55:15 | Equal Scale | 3.025 (0.030) | 2.711 (0.027) | 2.779 (0.028) | 2.782 (0.028) | 2.781 (0.028) | |
| Empirical SE | 50:40:10/30:55:15 | Unequal Scale | 3.051 (0.031) | 2.746 (0.027) | 2.818 (0.028) | 2.822 (0.028) | 2.827 (0.028) | |
| Model SE | 80:15:5/60:30:10 | Equal Scale | 2.703 (0.002) | 2.574 (0.009) | 2.434 (0.005) | 2.436 (0.004) | 2.476 (0.005) | |
| Model SE | 80:15:5/60:30:10 | Unequal Scale | 2.719 (0.002) | 2.584 (0.009) | 2.457 (0.004) | 2.459 (0.004) | 2.500 (0.005) | |
| Model SE | 60:35:5/40:50:10 | Equal Scale | 2.747 (0.002) | 2.498 (0.005) | 2.510 (0.003) | 2.512 (0.003) | 2.565 (0.004) | |
| Model SE | 60:35:5/40:50:10 | Unequal Scale | 2.757 (0.002) | 2.512 (0.004) | 2.526 (0.003) | 2.528 (0.003) | 2.588 (0.004) | |
| Model SE | 50:40:10/30:55:15 | Equal Scale | 3.052 (0.002) | 2.774 (0.004) | 2.831 (0.003) | 2.834 (0.003) | 2.854 (0.004) | |
| Model SE | 50:40:10/30:55:15 | Unequal Scale | 3.064 (0.002) | 2.783 (0.004) | 2.844 (0.003) | 2.848 (0.003) | 2.878 (0.004) | |
| MSE | 80:15:5/60:30:10 | Equal Scale | 8.273 (0.164) | 6.117 (0.140) | 5.846 (0.121) | 5.853 (0.121) | 5.832 (0.123) | |
| MSE | 80:15:5/60:30:10 | Unequal Scale | 8.649 (0.175) | 6.323 (0.165) | 6.081 (0.124) | 6.090 (0.125) | 6.053 (0.123) | |
| MSE | 60:35:5/40:50:10 | Equal Scale | 7.917 (0.153) | 6.260 (0.123) | 6.266 (0.123) | 6.275 (0.123) | 6.275 (0.127) | |
| MSE | 60:35:5/40:50:10 | Unequal Scale | 7.960 (0.163) | 6.345 (0.129) | 6.250 (0.127) | 6.259 (0.127) | 6.502 (0.131) | |
| MSE | 50:40:10/30:55:15 | Equal Scale | 9.228 (0.180) | 7.873 (0.158) | 7.795 (0.156) | 7.811 (0.156) | 7.765 (0.156) | |
| MSE | 50:40:10/30:55:15 | Unequal Scale | 9.426 (0.184) | 7.988 (0.155) | 7.985 (0.155) | 8.001 (0.156) | 8.305 (0.162) | |
| Power | 80:15:5/60:30:10 | Equal Scale | 0.813 (0.006) | 0.764 (0.006) | 0.809 (0.006) | 0.809 (0.006) | 0.812 (0.006) | |
| Power | 80:15:5/60:30:10 | Unequal Scale | 0.816 (0.005) | 0.766 (0.006) | 0.808 (0.006) | 0.807 (0.006) | 0.792 (0.006) | |
| Power | 60:35:5/40:50:10 | Equal Scale | 0.865 (0.005) | 0.856 (0.005) | 0.872 (0.005) | 0.872 (0.005) | 0.874 (0.005) | |
| Power | 60:35:5/40:50:10 | Unequal Scale | 0.863 (0.005) | 0.846 (0.005) | 0.866 (0.005) | 0.866 (0.005) | 0.825 (0.005) | |
| Power | 50:40:10/30:55:15 | Equal Scale | 0.771 (0.006) | 0.758 (0.006) | 0.787 (0.006) | 0.787 (0.006) | 0.790 (0.006) | |
| Power | 50:40:10/30:55:15 | Unequal Scale | 0.770 (0.006) | 0.756 (0.006) | 0.786 (0.006) | 0.785 (0.006) | 0.739 (0.006) | |
| $\boldsymbol{x}$ **= 30** |  |  |  |  |  |  |  | |
| Bias | 80:15:5/60:30:10 | Equal Scale | -1.238 (0.038) | 1.041 (0.058) | 0.137 (0.047) | 0.139 (0.047) | -0.008 (0.046) | |
| Bias | 80:15:5/60:30:10 | Unequal Scale | -1.181 (0.039) | 1.076 (0.058) | 0.173 (0.048) | 0.175 (0.048) | -0.238 (0.046) | |
| Bias | 60:35:5/40:50:10 | Equal Scale | -0.817 (0.039) | 1.144 (0.051) | 0.336 (0.046) | 0.332 (0.046) | -0.024 (0.043) | |
| Bias | 60:35:5/40:50:10 | Unequal Scale | -0.829 (0.039) | 1.062 (0.051) | 0.298 (0.046) | 0.292 (0.046) | -0.532 (0.043) | |
| Bias | 50:40:10/30:55:15 | Equal Scale | -0.446 (0.043) | 1.236 (0.054) | 0.310 (0.050) | 0.307 (0.050) | -0.046 (0.046) | |
| Bias | 50:40:10/30:55:15 | Unequal Scale | -0.384 (0.043) | 1.302 (0.054) | 0.351 (0.050) | 0.347 (0.050) | -0.532 (0.046) | |
| Coverage | 80:15:5/60:30:10 | Equal Scale | 92.6% (0.004) | 94.1% (0.003) | 94.5% (0.003) | 94.5% (0.003) | 94.6% (0.003) | |
| Coverage | 80:15:5/60:30:10 | Unequal Scale | 92.0% (0.004) | 93.7% (0.003) | 94.3% (0.003) | 94.3% (0.003) | 94.4% (0.003) | |
| Coverage | 60:35:5/40:50:10 | Equal Scale | 94.8% (0.003) | 93.5% (0.003) | 94.8% (0.003) | 94.7% (0.003) | 95.3% (0.003) | |
| Coverage | 60:35:5/40:50:10 | Unequal Scale | 93.9% (0.003) | 93.4% (0.004) | 94.3% (0.003) | 94.3% (0.003) | 94.6% (0.003) | |
| Coverage | 50:40:10/30:55:15 | Equal Scale | 95.1% (0.003) | 94.0% (0.003) | 95.0% (0.003) | 94.9% (0.003) | 95.4% (0.003) | |
| Coverage | 50:40:10/30:55:15 | Unequal Scale | 95.2% (0.003) | 93.5% (0.003) | 94.8% (0.003) | 94.8% (0.003) | 95.0% (0.003) | |
| Empirical SE | 80:15:5/60:30:10 | Equal Scale | 2.708 (0.027) | 4.118 (0.041) | 3.346 (0.033) | 3.348 (0.033) | 3.234 (0.032) | |
| Empirical SE | 80:15:5/60:30:10 | Unequal Scale | 2.756 (0.028) | 4.100 (0.041) | 3.388 (0.034) | 3.390 (0.034) | 3.266 (0.033) | |
| Empirical SE | 60:35:5/40:50:10 | Equal Scale | 2.734 (0.027) | 3.613 (0.036) | 3.228 (0.032) | 3.227 (0.032) | 3.019 (0.030) | |
| Empirical SE | 60:35:5/40:50:10 | Unequal Scale | 2.744 (0.027) | 3.623 (0.036) | 3.252 (0.033) | 3.250 (0.032) | 3.060 (0.031) | |
| Empirical SE | 50:40:10/30:55:15 | Equal Scale | 3.025 (0.030) | 3.847 (0.038) | 3.518 (0.035) | 3.516 (0.035) | 3.265 (0.033) | |
| Empirical SE | 50:40:10/30:55:15 | Unequal Scale | 3.051 (0.031) | 3.841 (0.038) | 3.523 (0.035) | 3.520 (0.035) | 3.281 (0.033) | |
| Model SE | 80:15:5/60:30:10 | Equal Scale | 2.703 (0.002) | 5.052 (0.067) | 3.384 (0.005) | 3.389 (0.005) | 3.275 (0.005) | |
| Model SE | 80:15:5/60:30:10 | Unequal Scale | 2.719 (0.002) | 4.984 (0.103) | 3.385 (0.005) | 3.389 (0.005) | 3.277 (0.005) | |
| Model SE | 60:35:5/40:50:10 | Equal Scale | 2.747 (0.002) | 3.825 (0.010) | 3.288 (0.004) | 3.288 (0.004) | 3.090 (0.004) | |
| Model SE | 60:35:5/40:50:10 | Unequal Scale | 2.757 (0.002) | 3.781 (0.009) | 3.281 (0.004) | 3.280 (0.004) | 3.104 (0.004) | |
| Model SE | 50:40:10/30:55:15 | Equal Scale | 3.052 (0.002) | 3.977 (0.008) | 3.565 (0.004) | 3.564 (0.004) | 3.317 (0.004) | |
| Model SE | 50:40:10/30:55:15 | Unequal Scale | 3.064 (0.002) | 3.975 (0.008) | 3.564 (0.004) | 3.562 (0.004) | 3.327 (0.004) | |
| MSE | 80:15:5/60:30:10 | Equal Scale | 8.865 (0.173) | 18.038 (0.564) | 11.210 (0.231) | 11.228 (0.232) | 10.454 (0.215) | |
| MSE | 80:15:5/60:30:10 | Unequal Scale | 8.991 (0.174) | 17.965 (0.493) | 11.506 (0.238) | 11.519 (0.238) | 10.718 (0.215) | |
| MSE | 60:35:5/40:50:10 | Equal Scale | 8.139 (0.159) | 14.363 (0.326) | 10.529 (0.212) | 10.519 (0.212) | 9.113 (0.178) | |
| MSE | 60:35:5/40:50:10 | Unequal Scale | 8.219 (0.163) | 14.250 (0.322) | 10.661 (0.220) | 10.643 (0.220) | 9.646 (0.196) | |
| MSE | 50:40:10/30:55:15 | Equal Scale | 9.347 (0.187) | 16.324 (0.365) | 12.471 (0.253) | 12.456 (0.253) | 10.663 (0.211) | |
| MSE | 50:40:10/30:55:15 | Unequal Scale | 9.455 (0.185) | 16.445 (0.347) | 12.530 (0.247) | 12.511 (0.247) | 11.045 (0.218) | |
| Power | 80:15:5/60:30:10 | Equal Scale | 0.813 (0.006) | 0.646 (0.007) | 0.786 (0.006) | 0.786 (0.006) | 0.801 (0.006) | |
| Power | 80:15:5/60:30:10 | Unequal Scale | 0.816 (0.005) | 0.659 (0.007) | 0.784 (0.006) | 0.785 (0.006) | 0.778 (0.006) | |
| Power | 60:35:5/40:50:10 | Equal Scale | 0.865 (0.005) | 0.833 (0.005) | 0.843 (0.005) | 0.843 (0.005) | 0.855 (0.005) | |
| Power | 60:35:5/40:50:10 | Unequal Scale | 0.863 (0.005) | 0.831 (0.005) | 0.841 (0.005) | 0.841 (0.005) | 0.818 (0.005) | |
| Power | 50:40:10/30:55:15 | Equal Scale | 0.771 (0.006) | 0.740 (0.006) | 0.724 (0.006) | 0.723 (0.006) | 0.751 (0.006) | |
| Power | 50:40:10/30:55:15 | Unequal Scale | 0.770 (0.006) | 0.739 (0.006) | 0.721 (0.006) | 0.720 (0.006) | 0.694 (0.007) | |
| ^a^ for truncated negative binomial distributions of productivity loss outcomes in the two arms;  OLS: ordinary least squares; NB: negative binomial; ZTNB: two-part model – logistic regression for the probability of being zero, and generalized linear regression with zero-truncated NB distribution for the non-zeros; ZG: two-part model – logistic regression for the probability of being zero, and generalized linear regression with Gamma distribution for the non-zeros; Three-part: multinomial logistic regression for the probabilities of being zero and 60 and generalized linear regression with Beta distribution for the those with values in (0, 60) (transformed to (0, 1)). | | | | | | | | |

**Supplementary Table S3. Performance measures for the number of observations each arm = 200.**

| **Measure** | **Distribution of productivity loss outcome** | **Scales in the two arms^a^** | **OLS** | **NB** | **ZTNB** | **ZG** | **Three-part** | |
| --- | --- | --- | --- | --- | --- | --- | --- | --- |
| $\boldsymbol{x}$ **= mean** |  |  |  |  |  |  |  | |
| Bias | 80:15:5/60:30:10 | Equal Scale | 0.016 (0.027) | 0.022 (0.028) | 0.007 (0.027) | 0.009 (0.027) | 0.041 (0.027) |  |
| Bias | 80:15:5/60:30:10 | Unequal Scale | -0.020 (0.027) | -0.012 (0.027) | -0.031 (0.027) | -0.029 (0.027) | -0.210 (0.027) |  |
| Bias | 60:35:5/40:50:10 | Equal Scale | -0.106 (0.027) | -0.053 (0.028) | -0.068 (0.028) | -0.065 (0.028) | -0.102 (0.027) |  |
| Bias | 60:35:5/40:50:10 | Unequal Scale | -0.088 (0.028) | -0.044 (0.028) | -0.057 (0.028) | -0.054 (0.028) | -0.551 (0.027) |  |
| Bias | 50:40:10/30:55:15 | Equal Scale | -0.062 (0.031) | 0.031 (0.031) | 0.007 (0.031) | 0.009 (0.031) | -0.026 (0.030) |  |
| Bias | 50:40:10/30:55:15 | Unequal Scale | -0.085 (0.031) | 0.006 (0.031) | -0.019 (0.031) | -0.017 (0.031) | -0.568 (0.030) |  |
| Coverage | 80:15:5/60:30:10 | Equal Scale | 94.7% (0.003) | 94.6% (0.003) | 94.7% (0.003) | 94.6% (0.003) | 94.8% (0.003) |  |
| Coverage | 80:15:5/60:30:10 | Unequal Scale | 95.5% (0.003) | 95.2% (0.003) | 95.3% (0.003) | 95.3% (0.003) | 95.4% (0.003) |  |
| Coverage | 60:35:5/40:50:10 | Equal Scale | 94.9% (0.003) | 94.8% (0.003) | 94.7% (0.003) | 94.7% (0.003) | 95.0% (0.003) |  |
| Coverage | 60:35:5/40:50:10 | Unequal Scale | 94.7% (0.003) | 94.6% (0.003) | 94.6% (0.003) | 94.6% (0.003) | 93.4% (0.004) |  |
| Coverage | 50:40:10/30:55:15 | Equal Scale | 94.8% (0.003) | 94.8% (0.003) | 95.0% (0.003) | 95.0% (0.003) | 94.9% (0.003) |  |
| Coverage | 50:40:10/30:55:15 | Unequal Scale | 94.8% (0.003) | 94.6% (0.003) | 94.7% (0.003) | 94.6% (0.003) | 93.8% (0.003) |  |
| Empirical SE | 80:15:5/60:30:10 | Equal Scale | 1.923 (0.019) | 1.967 (0.020) | 1.919 (0.019) | 1.920 (0.019) | 1.895 (0.019) |  |
| Empirical SE | 80:15:5/60:30:10 | Unequal Scale | 1.899 (0.019) | 1.943 (0.019) | 1.895 (0.019) | 1.896 (0.019) | 1.876 (0.019) |  |
| Empirical SE | 60:35:5/40:50:10 | Equal Scale | 1.937 (0.019) | 1.978 (0.020) | 1.947 (0.019) | 1.949 (0.019) | 1.890 (0.019) |  |
| Empirical SE | 60:35:5/40:50:10 | Unequal Scale | 1.972 (0.020) | 1.998 (0.020) | 1.976 (0.020) | 1.977 (0.020) | 1.933 (0.019) |  |
| Empirical SE | 50:40:10/30:55:15 | Equal Scale | 2.165 (0.022) | 2.210 (0.022) | 2.184 (0.022) | 2.186 (0.022) | 2.127 (0.021) |  |
| Empirical SE | 50:40:10/30:55:15 | Unequal Scale | 2.163 (0.022) | 2.207 (0.022) | 2.182 (0.022) | 2.184 (0.022) | 2.131 (0.021) |  |
| Model SE | 80:15:5/60:30:10 | Equal Scale | 1.913 (0.001) | 1.974 (0.002) | 1.910 (0.001) | 1.911 (0.001) | 1.884 (0.001) |  |
| Model SE | 80:15:5/60:30:10 | Unequal Scale | 1.921 (0.001) | 1.982 (0.002) | 1.921 (0.001) | 1.922 (0.001) | 1.896 (0.001) |  |
| Model SE | 60:35:5/40:50:10 | Equal Scale | 1.940 (0.001) | 1.978 (0.001) | 1.947 (0.001) | 1.948 (0.001) | 1.895 (0.001) |  |
| Model SE | 60:35:5/40:50:10 | Unequal Scale | 1.949 (0.001) | 1.988 (0.001) | 1.957 (0.001) | 1.959 (0.001) | 1.911 (0.001) |  |
| Model SE | 50:40:10/30:55:15 | Equal Scale | 2.154 (0.001) | 2.200 (0.001) | 2.171 (0.001) | 2.172 (0.001) | 2.118 (0.001) |  |
| Model SE | 50:40:10/30:55:15 | Unequal Scale | 2.164 (0.001) | 2.209 (0.001) | 2.181 (0.001) | 2.183 (0.001) | 2.133 (0.001) |  |
| MSE | 80:15:5/60:30:10 | Equal Scale | 3.699 (0.076) | 3.868 (0.079) | 3.683 (0.076) | 3.686 (0.076) | 3.593 (0.074) |  |
| MSE | 80:15:5/60:30:10 | Unequal Scale | 3.607 (0.072) | 3.775 (0.077) | 3.592 (0.072) | 3.596 (0.072) | 3.562 (0.072) |  |
| MSE | 60:35:5/40:50:10 | Equal Scale | 3.763 (0.074) | 3.914 (0.078) | 3.796 (0.075) | 3.801 (0.075) | 3.580 (0.070) |  |
| MSE | 60:35:5/40:50:10 | Unequal Scale | 3.895 (0.079) | 3.992 (0.081) | 3.908 (0.079) | 3.912 (0.079) | 4.039 (0.080) |  |
| MSE | 50:40:10/30:55:15 | Equal Scale | 4.689 (0.094) | 4.883 (0.097) | 4.770 (0.094) | 4.776 (0.095) | 4.524 (0.091) |  |
| MSE | 50:40:10/30:55:15 | Unequal Scale | 4.686 (0.096) | 4.872 (0.099) | 4.762 (0.097) | 4.768 (0.097) | 4.861 (0.100) |  |
| Power | 80:15:5/60:30:10 | Equal Scale | 0.981 (0.002) | 0.976 (0.002) | 0.981 (0.002) | 0.981 (0.002) | 0.984 (0.002) |  |
| Power | 80:15:5/60:30:10 | Unequal Scale | 0.984 (0.002) | 0.979 (0.002) | 0.982 (0.002) | 0.982 (0.002) | 0.982 (0.002) |  |
| Power | 60:35:5/40:50:10 | Equal Scale | 0.992 (0.001) | 0.990 (0.001) | 0.991 (0.001) | 0.991 (0.001) | 0.994 (0.001) |  |
| Power | 60:35:5/40:50:10 | Unequal Scale | 0.989 (0.001) | 0.987 (0.002) | 0.988 (0.002) | 0.988 (0.002) | 0.984 (0.002) |  |
| Power | 50:40:10/30:55:15 | Equal Scale | 0.969 (0.002) | 0.967 (0.003) | 0.969 (0.002) | 0.969 (0.002) | 0.975 (0.002) |  |
| Power | 50:40:10/30:55:15 | Unequal Scale | 0.966 (0.003) | 0.966 (0.003) | 0.967 (0.003) | 0.966 (0.003) | 0.951 (0.003) |  |
| $\boldsymbol{x}$ **= 0** |  |  |  |  |  |  |  | |
| Bias | 80:15:5/60:30:10 | Equal Scale | 1.051 (0.027) | -0.327 (0.024) | -0.056 (0.024) | -0.054 (0.024) | 0.047 (0.024) | |
| Bias | 80:15:5/60:30:10 | Unequal Scale | 1.015 (0.027) | -0.365 (0.024) | -0.095 (0.024) | -0.093 (0.024) | -0.173 (0.024) | |
| Bias | 60:35:5/40:50:10 | Equal Scale | 0.635 (0.027) | -0.582 (0.024) | -0.245 (0.025) | -0.238 (0.025) | -0.133 (0.025) | |
| Bias | 60:35:5/40:50:10 | Unequal Scale | 0.653 (0.028) | -0.585 (0.025) | -0.247 (0.025) | -0.239 (0.025) | -0.548 (0.025) | |
| Bias | 50:40:10/30:55:15 | Equal Scale | 0.358 (0.031) | -0.689 (0.027) | -0.211 (0.028) | -0.205 (0.028) | -0.093 (0.028) | |
| Bias | 50:40:10/30:55:15 | Unequal Scale | 0.334 (0.031) | -0.717 (0.027) | -0.239 (0.028) | -0.233 (0.028) | -0.591 (0.028) | |
| Coverage | 80:15:5/60:30:10 | Equal Scale | 91.7% (0.004) | 93.9% (0.003) | 94.6% (0.003) | 94.5% (0.003) | 95.0% (0.003) | |
| Coverage | 80:15:5/60:30:10 | Unequal Scale | 91.9% (0.004) | 94.7% (0.003) | 95.3% (0.003) | 95.3% (0.003) | 95.2% (0.003) | |
| Coverage | 60:35:5/40:50:10 | Equal Scale | 93.7% (0.003) | 92.9% (0.004) | 94.3% (0.003) | 94.3% (0.003) | 94.6% (0.003) | |
| Coverage | 60:35:5/40:50:10 | Unequal Scale | 93.3% (0.004) | 93.0% (0.004) | 94.4% (0.003) | 94.4% (0.003) | 93.7% (0.003) | |
| Coverage | 50:40:10/30:55:15 | Equal Scale | 94.7% (0.003) | 92.9% (0.004) | 94.6% (0.003) | 94.5% (0.003) | 94.9% (0.003) | |
| Coverage | 50:40:10/30:55:15 | Unequal Scale | 94.4% (0.003) | 93.3% (0.004) | 94.6% (0.003) | 94.6% (0.003) | 93.7% (0.003) | |
| Empirical SE | 80:15:5/60:30:10 | Equal Scale | 1.923 (0.019) | 1.688 (0.017) | 1.698 (0.017) | 1.700 (0.017) | 1.691 (0.017) | |
| Empirical SE | 80:15:5/60:30:10 | Unequal Scale | 1.899 (0.019) | 1.664 (0.017) | 1.675 (0.017) | 1.676 (0.017) | 1.672 (0.017) | |
| Empirical SE | 60:35:5/40:50:10 | Equal Scale | 1.937 (0.019) | 1.728 (0.017) | 1.758 (0.018) | 1.760 (0.018) | 1.747 (0.017) | |
| Empirical SE | 60:35:5/40:50:10 | Unequal Scale | 1.972 (0.020) | 1.744 (0.017) | 1.782 (0.018) | 1.784 (0.018) | 1.777 (0.018) | |
| Empirical SE | 50:40:10/30:55:15 | Equal Scale | 2.165 (0.022) | 1.940 (0.019) | 1.995 (0.020) | 1.998 (0.020) | 1.982 (0.020) | |
| Empirical SE | 50:40:10/30:55:15 | Unequal Scale | 2.163 (0.022) | 1.921 (0.019) | 1.987 (0.020) | 1.989 (0.020) | 1.987 (0.020) | |
| Model SE | 80:15:5/60:30:10 | Equal Scale | 1.913 (0.001) | 1.717 (0.003) | 1.706 (0.002) | 1.707 (0.002) | 1.712 (0.002) | |
| Model SE | 80:15:5/60:30:10 | Unequal Scale | 1.921 (0.001) | 1.721 (0.003) | 1.714 (0.002) | 1.715 (0.002) | 1.721 (0.002) | |
| Model SE | 60:35:5/40:50:10 | Equal Scale | 1.940 (0.001) | 1.729 (0.002) | 1.760 (0.002) | 1.761 (0.002) | 1.771 (0.002) | |
| Model SE | 60:35:5/40:50:10 | Unequal Scale | 1.949 (0.001) | 1.738 (0.002) | 1.771 (0.002) | 1.772 (0.002) | 1.786 (0.002) | |
| Model SE | 50:40:10/30:55:15 | Equal Scale | 2.154 (0.001) | 1.927 (0.002) | 1.982 (0.002) | 1.985 (0.002) | 1.986 (0.002) | |
| Model SE | 50:40:10/30:55:15 | Unequal Scale | 2.164 (0.001) | 1.938 (0.002) | 1.994 (0.002) | 1.996 (0.002) | 2.004 (0.002) | |
| MSE | 80:15:5/60:30:10 | Equal Scale | 4.803 (0.095) | 2.955 (0.062) | 2.887 (0.061) | 2.891 (0.061) | 2.860 (0.061) | |
| MSE | 80:15:5/60:30:10 | Unequal Scale | 4.636 (0.089) | 2.903 (0.059) | 2.815 (0.056) | 2.819 (0.056) | 2.826 (0.057) | |
| MSE | 60:35:5/40:50:10 | Equal Scale | 4.155 (0.083) | 3.325 (0.065) | 3.152 (0.062) | 3.154 (0.062) | 3.068 (0.061) | |
| MSE | 60:35:5/40:50:10 | Unequal Scale | 4.313 (0.086) | 3.384 (0.067) | 3.236 (0.066) | 3.239 (0.066) | 3.456 (0.068) | |
| MSE | 50:40:10/30:55:15 | Equal Scale | 4.813 (0.095) | 4.239 (0.084) | 4.025 (0.080) | 4.032 (0.080) | 3.936 (0.079) | |
| MSE | 50:40:10/30:55:15 | Unequal Scale | 4.790 (0.096) | 4.205 (0.086) | 4.004 (0.083) | 4.011 (0.083) | 4.298 (0.088) | |
| Power | 80:15:5/60:30:10 | Equal Scale | 0.981 (0.002) | 0.974 (0.002) | 0.982 (0.002) | 0.982 (0.002) | 0.982 (0.002) | |
| Power | 80:15:5/60:30:10 | Unequal Scale | 0.984 (0.002) | 0.976 (0.002) | 0.984 (0.002) | 0.983 (0.002) | 0.980 (0.002) | |
| Power | 60:35:5/40:50:10 | Equal Scale | 0.992 (0.001) | 0.990 (0.001) | 0.992 (0.001) | 0.992 (0.001) | 0.995 (0.001) | |
| Power | 60:35:5/40:50:10 | Unequal Scale | 0.989 (0.001) | 0.988 (0.002) | 0.990 (0.001) | 0.990 (0.001) | 0.985 (0.002) | |
| Power | 50:40:10/30:55:15 | Equal Scale | 0.969 (0.002) | 0.966 (0.003) | 0.977 (0.002) | 0.977 (0.002) | 0.979 (0.002) | |
| Power | 50:40:10/30:55:15 | Unequal Scale | 0.966 (0.003) | 0.965 (0.003) | 0.970 (0.002) | 0.970 (0.002) | 0.957 (0.003) | |
| $\boldsymbol{x}$ **= 30** |  |  |  |  |  |  |  | |
| Bias | 80:15:5/60:30:10 | Equal Scale | -1.156 (0.027) | 0.888 (0.039) | 0.174 (0.033) | 0.175 (0.033) | 0.068 (0.032) | |
| Bias | 80:15:5/60:30:10 | Unequal Scale | -1.193 (0.027) | 0.860 (0.038) | 0.140 (0.033) | 0.140 (0.033) | -0.222 (0.032) | |
| Bias | 60:35:5/40:50:10 | Equal Scale | -0.850 (0.027) | 0.977 (0.036) | 0.266 (0.032) | 0.260 (0.032) | -0.053 (0.030) | |
| Bias | 60:35:5/40:50:10 | Unequal Scale | -0.832 (0.028) | 1.003 (0.036) | 0.297 (0.033) | 0.290 (0.033) | -0.533 (0.031) | |
| Bias | 50:40:10/30:55:15 | Equal Scale | -0.370 (0.031) | 1.273 (0.038) | 0.384 (0.035) | 0.380 (0.035) | 0.039 (0.033) | |
| Bias | 50:40:10/30:55:15 | Unequal Scale | -0.394 (0.031) | 1.255 (0.038) | 0.365 (0.035) | 0.360 (0.035) | -0.543 (0.033) | |
| Coverage | 80:15:5/60:30:10 | Equal Scale | 90.4% (0.004) | 93.6% (0.003) | 94.6% (0.003) | 94.6% (0.003) | 94.9% (0.003) | |
| Coverage | 80:15:5/60:30:10 | Unequal Scale | 90.7% (0.004) | 93.9% (0.003) | 94.9% (0.003) | 94.9% (0.003) | 95.2% (0.003) | |
| Coverage | 60:35:5/40:50:10 | Equal Scale | 92.7% (0.004) | 93.0% (0.004) | 94.7% (0.003) | 94.7% (0.003) | 95.3% (0.003) | |
| Coverage | 60:35:5/40:50:10 | Unequal Scale | 92.6% (0.004) | 92.9% (0.004) | 94.4% (0.003) | 94.3% (0.003) | 93.8% (0.003) | |
| Coverage | 50:40:10/30:55:15 | Equal Scale | 94.7% (0.003) | 92.4% (0.004) | 94.7% (0.003) | 94.7% (0.003) | 95.2% (0.003) | |
| Coverage | 50:40:10/30:55:15 | Unequal Scale | 94.7% (0.003) | 91.7% (0.004) | 94.2% (0.003) | 94.3% (0.003) | 94.1% (0.003) | |
| Empirical SE | 80:15:5/60:30:10 | Equal Scale | 1.923 (0.019) | 2.741 (0.027) | 2.347 (0.023) | 2.347 (0.023) | 2.273 (0.023) | |
| Empirical SE | 80:15:5/60:30:10 | Unequal Scale | 1.899 (0.019) | 2.716 (0.027) | 2.325 (0.023) | 2.326 (0.023) | 2.251 (0.023) | |
| Empirical SE | 60:35:5/40:50:10 | Equal Scale | 1.937 (0.019) | 2.524 (0.025) | 2.280 (0.023) | 2.278 (0.023) | 2.130 (0.021) | |
| Empirical SE | 60:35:5/40:50:10 | Unequal Scale | 1.972 (0.020) | 2.535 (0.025) | 2.309 (0.023) | 2.307 (0.023) | 2.185 (0.022) | |
| Empirical SE | 50:40:10/30:55:15 | Equal Scale | 2.165 (0.022) | 2.698 (0.027) | 2.489 (0.025) | 2.487 (0.025) | 2.318 (0.023) | |
| Empirical SE | 50:40:10/30:55:15 | Unequal Scale | 2.163 (0.022) | 2.722 (0.027) | 2.495 (0.025) | 2.493 (0.025) | 2.315 (0.023) | |
| Model SE | 80:15:5/60:30:10 | Equal Scale | 1.913 (0.001) | 2.868 (0.008) | 2.345 (0.002) | 2.346 (0.002) | 2.275 (0.003) | |
| Model SE | 80:15:5/60:30:10 | Unequal Scale | 1.921 (0.001) | 2.863 (0.008) | 2.354 (0.002) | 2.355 (0.002) | 2.286 (0.003) | |
| Model SE | 60:35:5/40:50:10 | Equal Scale | 1.940 (0.001) | 2.572 (0.004) | 2.290 (0.002) | 2.288 (0.002) | 2.154 (0.002) | |
| Model SE | 60:35:5/40:50:10 | Unequal Scale | 1.949 (0.001) | 2.568 (0.004) | 2.296 (0.002) | 2.295 (0.002) | 2.171 (0.002) | |
| Model SE | 50:40:10/30:55:15 | Equal Scale | 2.154 (0.001) | 2.732 (0.003) | 2.490 (0.002) | 2.488 (0.002) | 2.319 (0.002) | |
| Model SE | 50:40:10/30:55:15 | Unequal Scale | 2.164 (0.001) | 2.735 (0.003) | 2.497 (0.002) | 2.495 (0.002) | 2.332 (0.002) | |
| MSE | 80:15:5/60:30:10 | Equal Scale | 5.036 (0.099) | 8.299 (0.200) | 5.537 (0.113) | 5.539 (0.113) | 5.172 (0.107) | |
| MSE | 80:15:5/60:30:10 | Unequal Scale | 5.029 (0.098) | 8.113 (0.193) | 5.426 (0.111) | 5.428 (0.111) | 5.114 (0.103) | |
| MSE | 60:35:5/40:50:10 | Equal Scale | 4.473 (0.086) | 7.322 (0.160) | 5.268 (0.107) | 5.258 (0.106) | 4.537 (0.089) | |
| MSE | 60:35:5/40:50:10 | Unequal Scale | 4.579 (0.092) | 7.431 (0.156) | 5.418 (0.109) | 5.406 (0.109) | 5.056 (0.100) | |
| MSE | 50:40:10/30:55:15 | Equal Scale | 4.822 (0.097) | 8.896 (0.181) | 6.340 (0.127) | 6.327 (0.127) | 5.375 (0.109) | |
| MSE | 50:40:10/30:55:15 | Unequal Scale | 4.834 (0.100) | 8.981 (0.185) | 6.357 (0.129) | 6.343 (0.129) | 5.653 (0.116) | |
| Power | 80:15:5/60:30:10 | Equal Scale | 0.981 (0.002) | 0.971 (0.002) | 0.975 (0.002) | 0.974 (0.002) | 0.978 (0.002) | |
| Power | 80:15:5/60:30:10 | Unequal Scale | 0.984 (0.002) | 0.975 (0.002) | 0.978 (0.002) | 0.978 (0.002) | 0.975 (0.002) | |
| Power | 60:35:5/40:50:10 | Equal Scale | 0.992 (0.001) | 0.989 (0.002) | 0.987 (0.002) | 0.987 (0.002) | 0.991 (0.001) | |
| Power | 60:35:5/40:50:10 | Unequal Scale | 0.989 (0.001) | 0.987 (0.002) | 0.985 (0.002) | 0.985 (0.002) | 0.981 (0.002) | |
| Power | 50:40:10/30:55:15 | Equal Scale | 0.969 (0.002) | 0.965 (0.003) | 0.955 (0.003) | 0.955 (0.003) | 0.961 (0.003) | |
| Power | 50:40:10/30:55:15 | Unequal Scale | 0.966 (0.003) | 0.964 (0.003) | 0.954 (0.003) | 0.955 (0.003) | 0.938 (0.003) | |
| ^a^ for truncated negative binomial distributions of productivity loss outcomes in the two arms;  OLS: ordinary least squares; NB: negative binomial; ZTNB: two-part model – logistic regression for the probability of being zero, and generalized linear regression with zero-truncated NB distribution for the non-zeros; ZG: two-part model – logistic regression for the probability of being zero, and generalized linear regression with Gamma distribution for the non-zeros; Three-part: multinomial logistic regression for the probabilities of being zero and 60 and generalized linear regression with Beta distribution for the those with values in (0, 60) (transformed to (0, 1)). | | | | | | | | |
